# Multi-compartment immune cell profiling highlights the prognostic relevance of CD127+ CD8+ T cells for patients with high-grade serous ovarian cancer

**DOI:** 10.3389/fimmu.2025.1607471

**Published:** 2025-10-27

**Authors:** Rebecca Rothe, Antje Tunger, Theresa Link, Xixi Lai, Jan Dominik Kuhlmann, Pauline Wimberger, Marc Schmitz, Rebekka Wehner

**Affiliations:** ^1^ National Center for Tumor Diseases (NCT), NCT/UCC Dresden, a partnership between DKFZ, Faculty of Medicine and University Hospital Carl Gustav Carus, TUD Dresden University of Technology, and Helmholtz-Zentrum Dresden-Rossendorf (HZDR), Dresden, Germany; ^2^ Institute of Immunology, Faculty of Medicine Carl Gustav Carus, TUD Dresden University of Technology, Dresden, Germany; ^3^ German Cancer Consortium (DKTK), Partner Site Dresden, Dresden, Germany; ^4^ Department of Gynecology and Obstetrics, University Hospital Carl Gustav Carus, TUD Dresden University of Technology, Dresden, Germany

**Keywords:** gynecologic cancer, immunological biomarker, immunophenotyping, liquid biopsy, local and peripheral immune system, tumor microenvironment

## Abstract

Ovarian cancer (OC) is a heterogeneous tumor entity with accumulated ascitic fluid in the peritoneal cavity, especially in advanced tumors. In general, a high immune cell infiltration has a favorable effect on OC patients’ outcomes. However, the composition of immune cells within the individual compartments of OC-associated locations may differ in their impact on patient prognosis. Therefore, we comprehensively investigated immune cell frequencies in matched peripheral blood, ascites, and tumor samples of 24 high-grade serous OC patients by flow cytometry and associated them with clinical parameters. Immune cell analysis demonstrated that the general immune cell infiltration was comparable between the three investigated compartments, with decreased proportions of CD8+ T cells in advanced stage OC. In addition, immune cell subsets varied significantly in their differentiation and phenotypic marker expression. In peripheral blood, classical monocytes, mature natural killer (NK) cells with cytotoxic potential (CD57+, CD16+, NKG2D+), and less differentiated T cells were more frequent. On the contrary, dendritic cells, and NKp46+ NK cells were prevalent in ascites. In OC tissues, high frequencies of immature neutrophils, CD16- NK cells, and effector memory T cells were found, although the intratumoral T cell frequency was significantly reduced compared to the two liquid samples. Additionally, T cell profiling showed high expression of one or multiple activating and inhibitory receptors in tumor samples. In particular, significant positive correlations of CD127+ CD8+ T cells among all three compartments were shown. Our results provide evidence that a higher proportion of peripheral CD127+ CD8+ T cells, which are memory T cells with low granzyme B production, was a prognostic biomarker for unfavorable progression-free survival of high-grade OC patients, independent of FIGO stage III/IV or residual tumor after surgery.

## 1 Introduction

Ovarian cancer (OC) is one of the most frequent gynecological cancer entities being the eighth common cause of mortality among malignant neoplasms in women worldwide (1, 2). Due to a lack of early symptoms, specific diagnostic molecular markers, and potent screening strategies, OC is mainly detected at a late stage resulting in a five-year survival rate below 30% for advanced tumors of FIGO stage III-IV (3–8). About 75% of all OC are histologically diagnosed as high-grade serous OC. This aggressive and fast-growing subtype arises especially from the epithelium of ovary or fallopian tubes and possesses a high metastatic potential (3, 5, 9, 10). In OC patients, the tumor microenvironment (TME) is highly heterogeneous with respect to different tumor-associated sites (4, 6, 11). Besides local immune responses of the individual TME, also peripheral blood (PB) immune cells affect anti-tumoral responses and immunotherapeutic efficacy (12). Further, tumor progression is accompanied by an accumulation of ascitic fluid in the peritoneal cavity enabling tumor cells to spread within the abdomen and settle metastases (11, 13). However, the microenvironment in ascites does not necessarily contain the same composition of immune cells as PB or tumor tissue and also metastatic lesions may differ (14, 15). Indeed, immune cell populations within the TME decisively influence therapy success and concomitantly overall survival (OS) of OC patients (16). Nevertheless, cancer cells can escape from immune surveillance. Therefore, a better understanding of the phenotypic immune cell composition in multiple OC-associated compartments could contribute to the derivation of possible prognostic biomarkers.

OC is an immunogenic tumor entity with tumor-infiltrated lymphocytes, especially CD8+ T cells, improving patients’ outcomes (17–20). Nevertheless, the TME of OC is supposed to be mainly immunosuppressive due to the presence of myeloid-derived suppressor cells (MDSC), neutrophils, immature or dysfunctional dendritic cells (DC), and regulatory T cells (T_reg_) (21, 22). For instance, MDSC subpopulations are able to promote tumor growth and progression by impairing immunity and inducing tumor immune evasion (3, 23). In this context, MDSC inhibit T cell proliferation, suppress CD8+ T cell- or natural killer (NK) cell-mediated tumor cell lysis, and promote the development of T_reg_ (4, 7, 21, 24). In the local TME of OC patients, a low NK cell infiltration is reported with CD16+ NK cells predicting unfavorable OS and CD56+ or CD57+ NK cells subsets improving OS (19). Further, mature DC are key regulators of the anti-tumoral immune response. DC initiate immune responses and promote cytotoxic activities of CD8+ T cells (25). However, tumor cells might affect DC development and activation escaping immune surveillance (26). Tumor-infiltrated plasmacytoid DC (pDC) contribute to an immunosuppressive TME resulting in an early relapse and poor prognosis of OC patients, potentially by upregulating T_reg_ infiltration (21, 27–30). However, OC cells negatively regulate the phenotype and effector function of infiltrated lymphocytes leading to an escape from anti-tumor immune responses. Thereby, tumor cells use several escape mechanisms including the expression of immune checkpoint molecules, like programmed death-ligand 1 (PD-L1), being associated with a unfavorable prognosis of OC patients (31, 32). In addition, sustained exposure to antigens or suppression by other immune cells of the TME direct T cells in an exhaustion state with reduced cytotoxic function (7, 11). Even therapeutic inhibition of one immune checkpoint is bypassed through the upregulation of other co-inhibitory receptors retaining immune escape (33). Thus, clinical trials investigating immune checkpoint inhibitor monotherapy or combination therapy with chemotherapeutics did not improve OC patients’ outcomes (34, 35).

Within the various OC-associated locations having their unique microenvironments, different immune cell populations can be both beneficial and detrimental in terms of the clinical outcome (3, 31, 36). Even multiple, differentially polarized TME may co-exist within the heterogeneous metastatic lesions of one patient as published in a case study by Jiménez-Sánchez et al. (37). In this context, previous investigations often led to contradictory conclusions as these studies mainly analyzed selected immune cell populations in one or two compartments, but a combined investigation of local and systemic immunity is needed (19). The variability of effector cell subtypes with divergent prognostic values among the OC-associated compartments makes it difficult to find reliable biomarkers when only one selected location is studied (38, 39). Thus, there are no immune-related prognostic biomarkers in clinical application. Currently, only serum cancer antigen 125 (CA125) is used as a biomarker in OC, but with limitations in specificity, sensitivity, and correlations regarding survival benefit (40). So far, OC studies focused on T cells (11) or other single immune cell subpopulations, like NK cells (17), MDSC (41), monocytes (42), and DC (43, 44), but studies with comprehensive immune cell phenotyping comparing multiple immune cell populations in several OC-associated locations are rare. Therefore, our study focused on the phenotypic characterization of various innate and adaptive immune cells in matched samples of PB, ascites, and tumor tissues to provide new insights in the complex OC microenvironments and contribute to the implementation of novel prognostic biomarkers.

## 2 Materials and methods

### 2.1 Patients and study design

This study included 28 treatment-naïve patients with high-grade serous OC undergoing primary debulking surgery in 2019-2024 (**Table 1**). Primary resected patients with grading G3 and FIGO stage III-IV were included in this comprehensive investigation. Prior to surgery, all patients were informed about the study and gave their consent for the examination of obtained samples at the University Hospital Carl Gustav Carus Dresden. The Ethics Committee of TUD Dresden University of Technology approved the retrospective analysis of clinical and biological data (EK44022018, EK74032013). PB (n = 23, median = 16 ml, range = 9–18 ml) was collected in EDTA-containing monovettes. Intraoperatively, fresh tumor tissues resected from peritoneal carcinomatosis herds and ovarian tissue (n = 24, median = 1.42 g, range = 0.03-18.1 g), as well as ascites (n = 21, median = 110 ml, range = 10–440 ml) were taken and processed as described in the following sections. Of 24 patients, at least two matched samples of different OC-associated compartments (PB, ascites, and tumor tissue) were obtained forming the main cohort of this study. Furthermore, PB samples were received from four additional patients with the same patient criteria and immune cell profiles as the patients in the main cohort. Therefore, they were included in the association analyses of immune cell frequencies with clinical parameters in order to expand the patient number.

**Table 1:** Characteristics of OC patients.

	n = 28
Age	
Median (years)	69
Range (years)	47 – 80
Serum CA125 at surgery	
Median (IU/ml)	487.5
Range (IU/ml)	28.6 – 29800
FIGO stage	
III IIIb IIIc	19 (67.9%) 4 (21.1%) 15 (78.9%)
IV IVa IVb	9 (32.1%) 1 (11.1%) 8 (88.9%)
Grading	
G3	28 (100%)
Histology	
Serous	27 (96.4%)
Clear cell	1 (3.6%)
Mutation status	
mutation BRCA1 BRCA2 ATM RAD51C no mutation unknown	11 (39.3%) 3 (27.3%) 5 (45.5%) 2 (18.2%) 1 (9.1%) 15 (53.6%) 2 (7.1%)
Surgical outcome	
Macroscopic complete resection	22 (78.6%)
Any residual tumor	6 (21.4%)
Progression	2-year follow up: n = 16
Relapse	9 (50.0%)
No relapse	7 (38.9%)
Survival	2-year follow up: n = 18
Dead	6 (33.3%)
Alive	12 (66.7%)

Moreover, immune cell frequencies of PB samples from OC patients were compared to PB samples from gender- and age-matched healthy donors (HD; median: 66 years, range: 60–72 years). Buffy coats from female blood donors were received from the German Red Cross blood donation center (Dresden, Germany; EK138042014) with the consent of the donors.

### 2.2 Preparation of peripheral blood, ascites, and tumor tissue samples

Peripheral blood mononuclear cells (PBMCs) were isolated from PB samples by density gradient centrifugation (980 × g, 20 min) at room temperature (RT) using Pancoll^®^ (PAN-Biotech, Aidenbach, Germany). Hereinafter, PBMCs were harvested from the interphase, washed with cold phosphate buffered saline (PBS, Sigma-Aldrich, St. Louis, Missouri, United States), and passed over a 40 µm cell strainer (Falcon™, Thermo Fisher Scientific, Schwerte, Germany).

Further, ascites samples were centrifuged several times. First, the whole ascites samples were centrifuged at 260 × g for 10 min (RT) to eliminate larger debris fragments and aggregates. Resultant pellet was discarded and the supernatant was centrifuged again at 1800 × g for 10 min (RT). Hereinafter, the resultant supernatant was discarded and the pelleted cells contained the immune cells.

For immune cell detection in tumor tissues, the samples were manually minced with a scalpel and further enzymatically processed using Tumor Dissociation Kit and gentleMACS™ Dissociator (both from Miltenyi Biotec, Bergisch Gladbach, Germany). After digestion according to the standard protocol (1 h, 37 °C), the suspension was filtered through a 70 µm cell strainer (Falcon™, Thermo Fisher Scientific) and washed with Dulbecco’s Modified Eagle Medium (DMEM cell culture media, Gibco™, Thermo Fisher Scientific).

In case of remaining red blood cells in the pellets, patient samples were additionally incubated in ammonium-chloride-potassium lysing buffer (ACK buffer, Gibco™, Thermo Fisher Scientific) for 10 min at RT and washed with PBS. The pelleted immune cells were counted in acetic acid (5%, Merck KGaA, Darmstadt, Germany) or trypan blue (0.4%, Gibco™, Thermo Fisher Scientific) using the Neubauer counting chamber (Marienfeld GmbH & Co.KG, Lauda Königshofen, Germany). For subsequent staining procedure, at least 3 × 10^5^ cells (blood) or 1 × 10^6^ cells (ascites and tumor tissue) for each individual staining panel were used. Thus, depending on the count of isolated PBMCs from each patient sample, different quantities of immune cell panels were stained and analyzed.

### 2.3 Antibody staining and flow cytometric analysis

For specific staining of surface antigens, the cells were incubated with appropriate antibodies in the dark for 15 min at 4 °C (**Supplementary Table 1**, antibody dilution in brilliant stain buffer, BD Biosciences, Franklin Lakes, New Jersey, United States). Hereinafter, the samples were washed with PBS (360 × g, 3 min, 4 °C) or further treated to stain intracellular markers. In terms of FoxP3 staining, cells were fixed and permeabilized using FoxP3 staining buffer set according to manufacturer’s instructions (Miltenyi Biotec) followed by an incubation step (30 min, 4 °C, dark) with fluorophore-labeled anti-human FoxP3 antibody or isotype control (**Supplementary Table 1**). On the other hand, cells were incubated with 4% paraformaldehyde solution (PFA, 10 min, on ice, dark, Sigma-Aldrich) and 0.1% saponin solution (3 min, 4 °C, dark, Carl Roth GmbH + Co. KG, Karlsruhe, Germany) for the intracytoplasmic staining of myeloperoxidase (MPO) and arginase 1 in the neutrophil staining panel (antibody dilution in saponin buffer). Prior to the flow cytometric measurement using flow cytometer BD LSR Fortessa™ and BD FACSDiva™ software (BD Biosciences), samples were filtered using nylon membranes (Sefar, Thal, Switzerland) and lastly 7-AAD (BD Biosciences) was added to stain for cell viability.

FlowLogic™ software (version 8.6; Inivai Technologies Pty. Ltd., Mentone Victoria, Australia) was used for data evaluation. For each sample, isotype control approaches served as controls for particular gating strategies (**Supplementary Figures 1**–**5**). Hierarchical gating comprised duplicate exclusion (FSC-A vs. FSC-H) followed by dead cell exclusion (SSC-A vs. 7-AAD staining) and specific antigen stainings. Single patient samples, where fluorescence intensities of the marker proteins to be detected did not correspond to the generally valid gating strategy for all OC patients of the respective panel, failed quality control and were excluded from the respective analysis. Boolean functions were applied to the gating strategies in FlowLogic™ in terms of i) expression or absence of CD10 and CD16 by neutrophils, ii) expression or absence of CD4 and CD8 by γδT cells, and iii) phenotypic characterization of T cells with respect to the expression of several co-activating and co-inhibitory receptor combinations.

### 2.4 Granzyme B production of CD127+ CD8+ T cells from frozen PBMC samples

In order to further characterize CD127+ CD8+ T cells, frozen PBMC samples of 13 OC patients were used. After thawing the cells in PBS supplemented with 4% sodium citrate and 0.5% human serum, PBMCs were placed in RPMI 1640 medium (Gibco™, Thermo Fisher Scientific) with 10% human serum (C.C.Pro GmbH, Neustadt, Deutschland), 200 mM L-glutamine (Sigma-Aldrich), 100 mM sodium pyruvate (Sigma-Aldrich), 1% non-essential amino acids (Gibco™, Thermo Fisher Scientific), 100 µg/ml penicillin, and 100 µg/ml streptomycin (both Gibco™, Thermo Fisher Scientific) in 96 well U-bottom plates (3 × 10^5^ cells per well) and were stimulated by Phorbol-12-myristate-13-acetate (PMA; 10 ng/ml, Sigma-Aldrich) and ionomycin (1 µg/ml, Sigma-Aldrich). To block exocytosis of cytokines, brefeldin A (1 µg/ml, Sigma-Aldrich) was added simultaneously to the cell culture. After 4 h incubation (36 °C, humidified atmosphere containing 5% CO_2_), the cells were harvested and stained with fixable viability stain 575V (FVS, BD Biosciences) according to manufacturer’s instructions to discriminate between viable and dead cells in the flow cytometric analysis. Further, cells were stained with antibodies directed against surface antigens (CD3, CD8, CD127, CCR7, CD45RA, **Supplementary Table 1**, diluted in Brilliant Stain Buffer, BD Biosciences) for 15 min at 4 °C. After washing, cells were fixed with ice-cold 4% PFA (Merck) for 15 min and permeabilized with 0.1% saponin (Merck) for 3 min at 4 °C. Subsequently, staining of intracellular granzyme B (GrzB, **Supplementary Table 1**, diluted in 0.1% saponin buffer) with an APC-labeled antibody followed for 15 min at 4 °C. Prior to flow cytometric analysis using LSR Fortessa™ and BD FACSDiva™ software (BD Biosciences), samples were filtered through nylon membranes (Sefar, Thal, Switzerland). FlowLogic™ software (version 8.6; Inivai Technologies Pty. Ltd., Mentone Victoria, Australia) was used for data evaluation. For each sample, isotype control approaches served as controls for particular gating strategies (**Supplementary Figure 6**). Hierarchical gating comprised duplicate exclusion (FSC-A vs. FSC-H) followed by dead cell exclusion (SSC-A vs. FVS staining) and specific antigen stainings.

### 2.5 Statistical analysis

All data sets were statistically analyzed and illustrated using GraphPad Prism 10.2.3 software (GraphPad Prism Inc., Boston, Massachusetts, United States). Dot plots show mean ± SEM and multivariate Cox proportional hazards regression model includes hazard ratios (HR) with 95% CI and corresponding p-values. Depending on the underlying data set, unpaired or paired t-test, one-way or two-way ANOVA with Bonferroni *post-hoc* test for multiple comparisons, and Log-rank test for Kaplan-Meier survival analysis were performed. In terms of correlation analysis, Pearson correlation coefficients and two-tailed p-vales were calculated. Values were considered significant according to significance levels of p ≤ 0.05 (*), p ≤ 0.01 (**), p < 0.001 (***). Statistical tests and significance levels are mentioned in the respective figure legends.

## 3 Results

**Figure 1:**
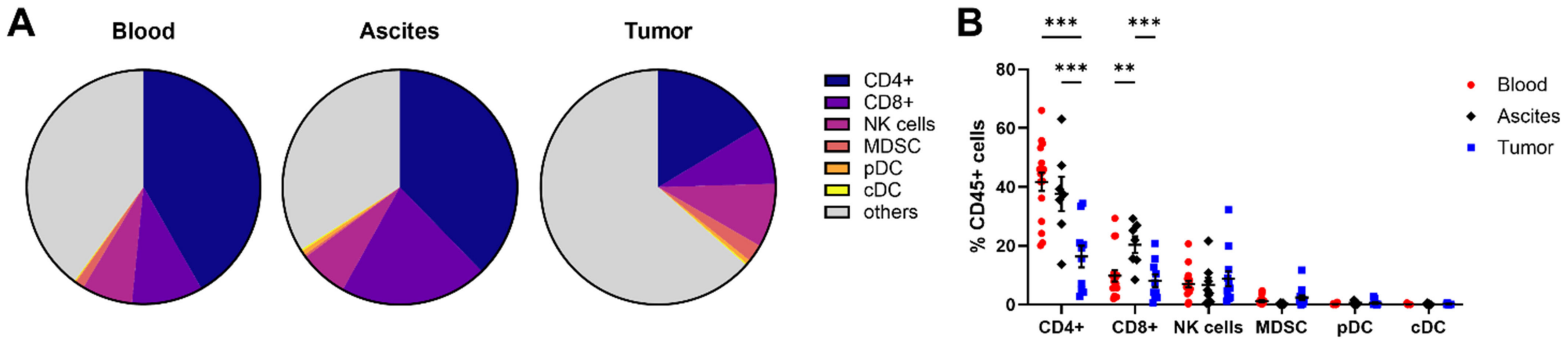
Overview of immune cell composition in matched peripheral blood, ascites, and tumor tissue samples. **(A)** Pie charts illustrate the mean proportions of immune cell subsets measured in all three OC-associated compartments in percentage of the CD45+ cell population. **(B)** Dot plot depicts immune cell frequencies of CD4+ and CD8+ T cells, NK cells, MDSC, pDC, as well as cDC for peripheral blood, ascites, and tumor tissues for individual patients. Mean ± SEM, two-way ANOVA, Bonferroni *post-hoc* test, **p ≤ 0.01, ***p < 0.001.

To get a comprehensive picture of immune cell frequencies and marker expression profiles in patients with high-grade serous OC, we performed multi-compartment immunophenotyping by flow cytometry using matched PB, ascites, and tumor tissue samples of 24 OC patients. For a first overview, we summarized the proportions of immune cell subsets measured in all three OC-associated compartments based on whole CD45+ cell population (**Figure 1**). Although the general composition is the same, the percentages of single populations differ in PB, ascites, and tumor tissue samples. CD4+ T cells represent the most frequent immune cell population followed by CD8+ lymphocytes, NK cells, MDSC, pDC and conventional DC (cDC). Thereby, tumor tissue contained significantly lower amounts of CD4+ T cells compared to both liquid samples, whereas the proportion of ascites-derived CD8+ T cells was significantly higher than PB and tumor tissue (**Figure 1B**). The proportions and phenotypes of the immune cells are described in more detail together with CD14+ monocytes, neutrophils, and γδT cells in the next chapters.

### 3.1 Immunophenotyping of various innate immune cell populations

**Figure 2:**
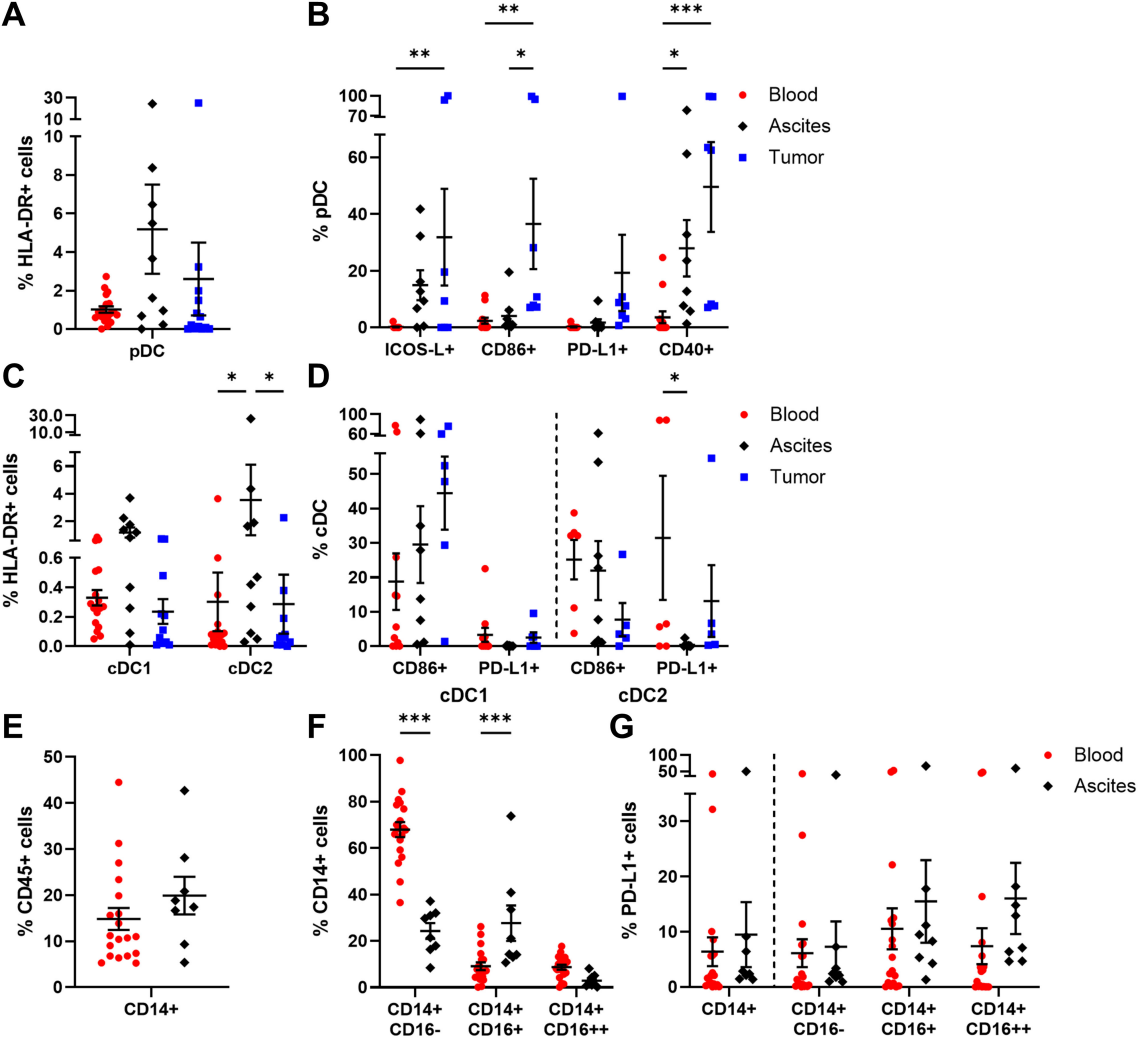
Characterization of DC and monocyte subpopulations in matched peripheral blood, ascites, and tumor tissue samples. Dot plots show the proportions of **(A)** pDC and **(B)** their expressed markers as well as **(C)** cDC1, cDC2, and **(D)** the analyzed marker molecules. For peripheral blood and ascites samples, the proportion of **(E)** monocytes, **(F)** monocyte subsets according to CD16 expression, and **(G)** their PD-L1 expression is presented. Mean ± SEM, two-way ANOVA, Bonferroni *post-hoc* test, *p ≤ 0.05, **p ≤ 0.01, ***p < 0.001.

In this section, a general overview of innate immune cells is provided entailing DC, monocytes, neutrophils, MDSC, and NK cells. We investigated pDC (CD303+ CD123+ HLA-DR+) and cDC (CD11c+ HLA-DR+) subpopulations, with cDC being further subdivided in cDC1 (CD141+) and cDC2 (CD1c+). Among the investigated OC compartments, pDC frequency was highest in ascites followed by tumor samples, with average percentages of 5.18 ± 2.32% and 2.60 ± 1.90% of HLA-DR+ cells, respectively, but without reaching significant difference compared to PB (1.01 ± 0.16% of HLA-DR+ cells, **Figure 2A**). Regarding the expression of additional surface proteins, especially tumor-infiltrated and to a lesser extent ascites-derived pDC expressed ICOS-L, CD86, and CD40 indicating higher pDC activity in OC tissue (**Figure 2B**). In contrast, the mentioned marker proteins were almost absent on PB-derived pDC. Furthermore, PD-L1+ pDC were increased in OC tissues without reaching significance compared to blood and ascites samples. The highest proportions of cDC1 and cDC2 were detected in ascites with 1.19 ± 0.37% and 3.55 ± 2.56% of HLA-DR+ cells, respectively (**Figure 2C**). Regarding their phenotypes, CD86+ cDC1 were more frequently detected than CD86+ cDC2 in tumor tissue, with 44.44 ± 10.62% and 7.72 ± 4.84%, respectively (**Figure 2D**, **Supplementary Figure 7**). For both liquid samples, CD86+ cDC1 and CD86+ cDC2 frequencies varied between 18-30% on average. Whereas PD-L1 expression on cDC1 was almost absent (< 4% of cDC1), PD-L1+ cDC2 were detected in PB and tumor tissue in a few OC patients.

Further, PB and ascites samples were analyzed in terms of monocyte abundance resulting in similar proportions, with 14.83 ± 2.38% and 19.91 ± 4.07% of CD45+ cells, respectively (**Figure 2E**). Additionally, the frequencies of several monocyte subpopulations classified as CD14+ CD16- (classical), CD14+ CD16+ (intermediate), and CD14+ CD16++ (non-classical) monocytes were studied (**Figure 2F**). Classical monocytes occurred predominantly in blood samples (blood: 67.94 ± 3.25% of CD14+ cells, ascites: 24.27 ± 3.43% of CD14+ cells), whereas intermediate monocytes were significantly enriched in ascites (blood: 9.13 ± 1.64% of CD14+ cells, ascites: 27.69 ± 7.61% of CD14+ cells). In turn, non-classical monocytes represented the least common subtype in both samples. On average, less than one fifth of the monocyte subsets expressed PD-L1 on their surfaces, with no statistical differences between the investigated liquid samples (**Figure 2G**). In comparison to HD, OC patients showed a significantly increased frequency of CD14+ monocytes in PB as well as numerically higher amounts of PD-L1+ monocytes and PD-L1+ monocyte subpopulations (**Supplementary Figure 8**).

**Figure 3:**
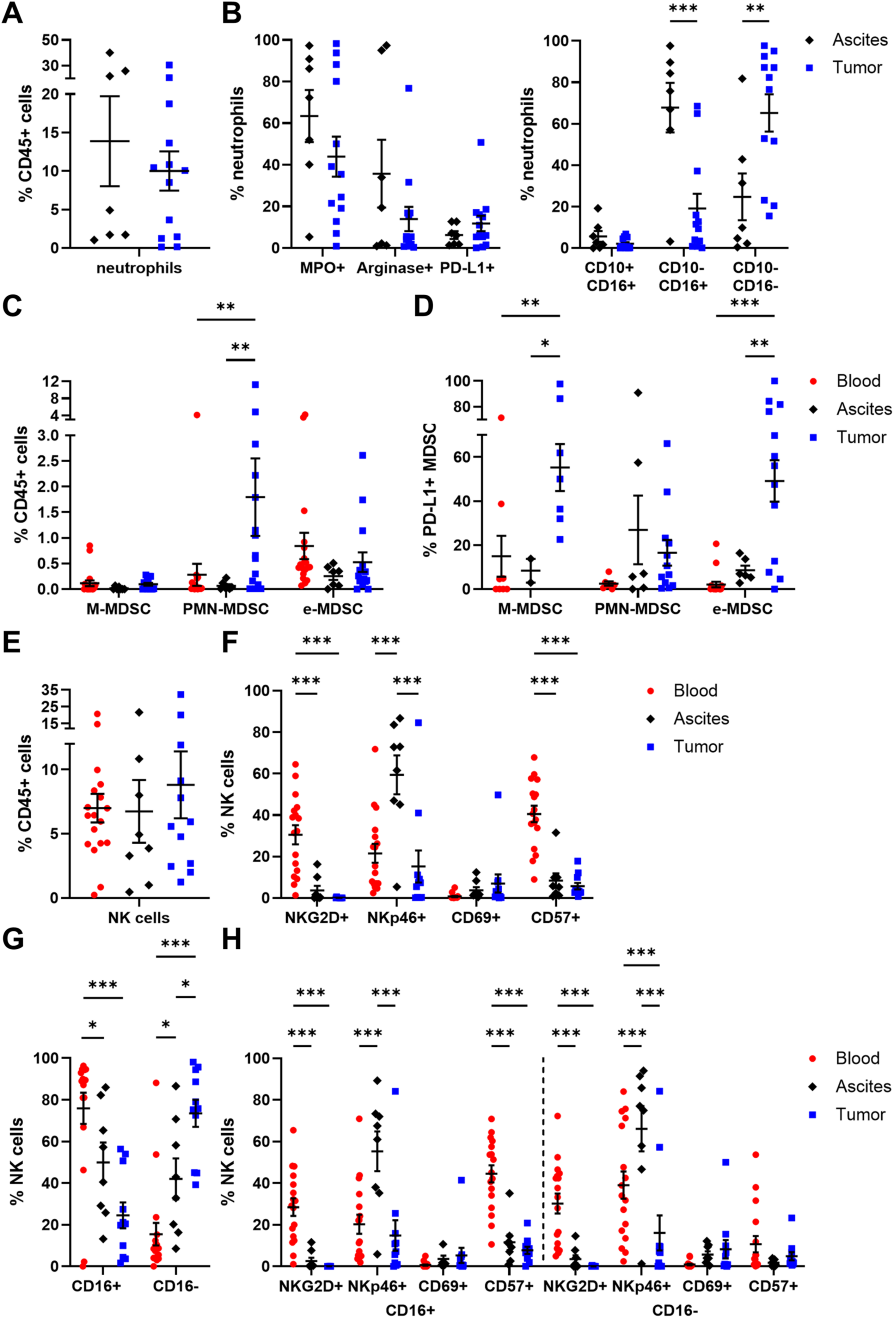
Characterization of neutrophils, MDSC, and NK cells in matched peripheral blood, ascites, and tumor tissue samples. Dot plots show the proportion of **(A)** neutrophils and **(B)** their expressed markers (left) as well as neutrophil maturation (right; immature: CD10- CD16-; mature: CD10-/+ CD16+) calculated by Boolean gating in FlowLogic™ using AND or AND NOT function. The proportion of **(C)** MDSC subsets and **(D)** their PD-L1 expression is presented for matched OC-associated sites. Similarly, proportion of **(E)** NK cells and **(F)** the analyzed marker molecules as well as **(G)** proportion of CD16+ and CD16- NK cell subpopulations and **(H)** their expressed markers are depicted in dot plots. Mean ± SEM, two-way ANOVA, Bonferroni *post-hoc* test, *p ≤ 0.05, **p ≤ 0.01, ***p < 0.001.

Regarding neutrophil (CD15+ CD66b+ CD11b+) abundance, no significant differences among ascites- or tumor tissue-derived neutrophils were obvious, with 13.88 ± 5.84% and 10.01 ± 2.54% of CD45+ cells, respectively (**Figure 3A**). Of note, neutrophils were studied only in ascites and tumor tissue samples of OC patients, as the PB sample preparation by density gradient centrifugation excluded neutrophils from PBMCs. Looking at the neutrophil phenotypes in more detail, PD-L1 was expressed in < 12% of neutrophils on average (**Figure 3B**). In terms of neutrophil activation, proportions of MPO+ and arginase 1+ neutrophils were higher in ascites than in tumor, with a 1.4- to 2.6-fold change, respectively. According to the literature, CD16low/- CD10- neutrophils are referred to be immature neutrophils, whereas CD16high CD10low/+ neutrophils reflect the mature phenotype (45–48). In our study, CD10- CD16+ mature neutrophils were significantly more prominent in ascites (ascites: 67.82 ± 11.94% of neutrophils, tumor: 19.15 ± 7.06% of neutrophils), while CD10- CD16- immature neutrophils dominated in OC tissues (ascites: 24.74 ± 11.28% of neutrophils, tumor: 65.19 ± 8.99% of neutrophils). Similarly, mature neutrophils in ascites co-expressed both maturation marker CD10 and CD16 two times more frequently (5.63 ± 2.62% of neutrophils) than their counterparts in tumor samples (2.10 ± 0.76% of neutrophils).

In addition, MDSC were distinguished in three subpopulations (49): CD14+ CD15- CD11b+ HLA-DR- (M-MDSC), CD14- CD15+ CD11b+ HLA-DR- (PMN-MDSC), and CD14- CD15- HLA-DR- CD33+ (e-MDSC). A significantly higher proportion of PMN-MDSC was found in tumor tissues (1.80 ± 0.76% of CD45+ cells) compared to blood and ascites samples (**Figure 3C**). In contrast, percentages of M-MDSC and e-MDSC were similar for all investigated OC-associated compartments, with < 1% of CD45+ cells. Interestingly, on average every second tumor-infiltrated M-MDSC and e-MDSC displayed PD-L1 expression (PD-L1+ M-MDSC: 55.26 ± 10.70%, PD-L1+ e-MDSC: 49.17 ± 9.40%) being significantly elevated in comparison to both liquid samples (blood PD-L1+ M-MDSC: 14.96 ± 9.32%, ascites PD-L1+ M-MDSC: 8.38 ± 5.41%, blood PD-L1+ e-MDSC: 2.09 ± 1.27%, ascites PD-L1+ e-MDSC: 8.56 ± 2.16%, **Figure 3D**).

In all investigated compartments, up to 10% of NK cells (CD3- CD56+) among CD45+ immune cells were detected (**Figure 3E**). A more detailed phenotypic characterization regarding the expression of NKG2D, NKp46, CD69, and CD57 showed that NKG2D and CD57 were mainly expressed by blood-derived NK cells, with 30.49 ± 4.60% and 40.53 ± 3.94%, respectively (**Figure 3F**), whereas NKp46 was found significantly more often on ascites-derived NK cells (59.40 ± 9.43% of NK cells). CD69 was the least expressed marker by NK cells in all three compartments. With regard to CD16 expression, defining two functional distinct NK cell subsets, significant differences between the individual compartments were evident as 75.91 ± 7.47% of blood-circulating NK cells carried this Fc receptor followed by NK cells in ascites (49.93 ± 9.54% of NK cells) and tumor-infiltrated NK cells (24.49 ± 6.18% of NK cells, **Figure 3G**). Both CD16+ and CD16- NK cell subpopulations displayed similar distributions of NKG2D, NKp46, CD69, and CD57 expression in all OC-associated compartments (**Figure 3H**). However, in PB samples, significantly higher proportions of CD57+ CD16+ NK cells and NKp46+ CD16- NK cells were observed in comparison to their respective counterparts (**Supplementary Figure 9**). Comparing NK cell subsets among HD and OC, a significantly reduced frequency of NKG2D+ NK cells and a numerically increased amount of NKp46+ NK cells in OC, especially in CD16+ NK cell subpopulations, was found (**Supplementary Figure 10**).

Summarizing the phenotyping of innate immune cell populations in multiple compartments of high-grade serous OC patients, we found lower proportions of classical monocytes and mature NK cells with cytotoxic potential in ascites and tumor than PB. Ascites samples showed elevated percentages of cDC being key regulators of immune responses, mature neutrophils (CD10- CD16+), and NKp46+ NK cells potentially exerting antitumoral effects. On the contrary, immature neutrophils (CD10- CD16-), PMN-MDSC, PD-L1+ M-MDSC, and PD-L1+ e-MDSC, pDC expressing ICOS-L, CD86, or CD40 as well as CD16- NK cell proportions with limited antitumoral potential dominated in tumor tissues.

### 3.2 Distribution and maturation of αβ T cell and γδT cell subsets in OC patient samples

Since tumor-infiltrated T lymphocytes are supposed to decisively affect clinical outcomes of high-grade serous OC patients (39), we focused on their differentiation stages and (co-)expression of functional markers in our study. Of note, αβT cells were studied in all three OC-associated compartments, whereas γδT cells were investigated only in ascites and tumor tissue samples, as γδT cell frequency is reported to be less frequent in blood samples (50).

**Figure 4:**
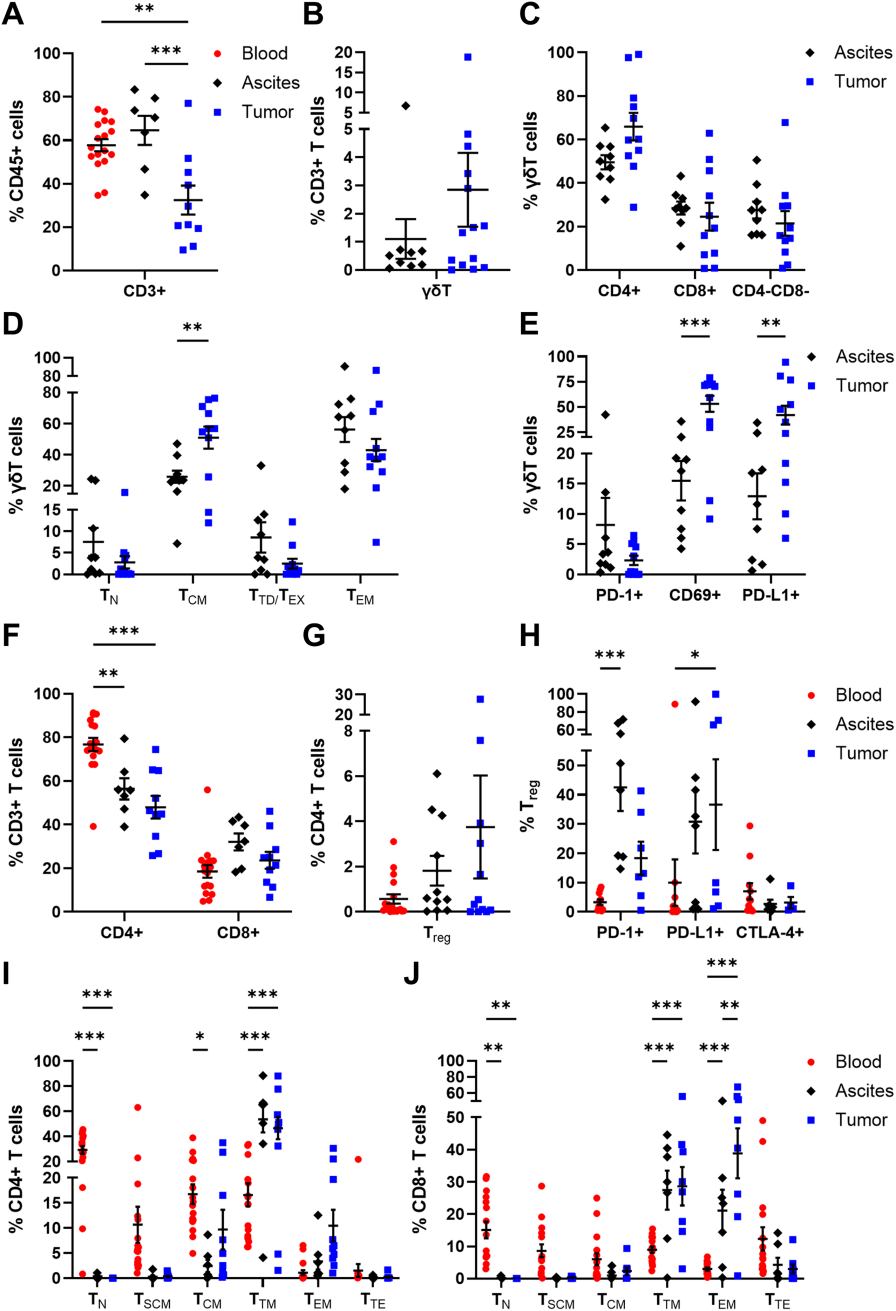
Distribution and maturation of γδT cells and αβT cell subpopulations in matched peripheral blood, ascites, and tumor tissue samples. Dot plots show the proportions of **(A)** CD3+ T cells, **(B)** γδT cells, and **(C)** γδT cell subsets according to CD4 and CD8 expression calculated by Boolean gating in FlowLogic™ using AND function, **(D)** their differentiation stages, and **(E)** their inhibitory marker expression. Further, dot plots present the proportions of **(F)** CD4+ and CD8+ αβT cells, **(G)** T_reg_ and **(H)** T_reg_ inhibitory marker molecules, as well as **(I)** CD4+ T cell and **(J)** CD8+ T cell differentiation stages. Mean ± SEM, ordinary one-way ANOVA **(A)** or two-way ANOVA **(D–F, H–J)**, Bonferroni *post-hoc* test, *p ≤ 0.05, **p ≤ 0.01, ***p < 0.001.

Proportions of CD3+ T cells (32.45 ± 6.66% of CD45+ cells) in OC tissues were significantly lower compared to blood (57.67 ± 2.80% of CD45+ cells) and ascites samples (64.54 ± 6.73% of CD45+ cells, **Figure 4A**). Among CD3+ T cells, γδT cells were detected in low percentages in ascites (1.10 ± 0.71% of CD3+ T cells) and tumor tissue samples (2.85 ± 1.31% of CD3+ T cells, **Figure 4B**). In addition, γδT cells were subdivided according to CD4 and CD8 expression pattern (11), whereby a CD4+ phenotype was more abundant in ascites and tumor samples, with > 50% of γδT cells each (**Figure 4C**). Likewise, CD8+ γδT cells and CD4- CD8- γδT cells displayed similar percentages in both investigated compartments. Further, γδT cells were distinguished into four subpopulations with respect to their differentiation stages based on CD45RA and CD27 expression (51, 52): T_N_ (naïve γδT cells: CD45RA+ CD27+), T_CM_ (central memory γδT cells: CD45RA- CD27+), T_TD/EX_ (terminally differentiated exhausted γδT cells: CD45RA+ CD27-), and T_EM_ (effector memory γδT cells: CD45RA- CD27-). Both memory γδT cell subsets (T_CM_ and T_EM_) represented the most frequently occurring ones in both investigated compartments (**Figure 4D**), with γδT_CM_ being significantly enriched in tumor tissues (50.95 ± 7.04% of γδT cells) and γδT_EM_ being more abundant in ascites samples (56.19 ± 8.04% of γδT cells) than in tumor samples (42.99 ± 7.14% of γδT cells). Moreover, significantly high proportions of tumor-infiltrated CD69+ γδT cells and PD-L1+ γδT cells were detected being at least 3-times more abundant than ascites-derived γδT cell counterparts (**Figure 4E**).

Besides γδT cells, the frequencies of αβT cell populations among CD3+ T cells were analyzed. In all investigated OC-associated compartments, CD4+ T cells displayed higher percentages than CD8+ T cells (**Figure 4F**), with a significantly increased proportion of blood-circulating CD4+ T cells (76.77 ± 3.03% of CD3+ T cells) and least tumor-infiltrated CD4+ T cells (47.99 ± 5.20% of CD3+ cells). Within the CD4+ T cell population, T_reg_ (CD4+ FoxP3+ CD25+ CD127-) are a special subset eliciting immunosuppressive effects. On average, T_reg_ proportions tended to increase from blood to ascites to tumor samples, although T_reg_ frequency was < 4% of CD4+ T cells (**Figure 4G**). Regarding the expression of immune checkpoint molecules, T_reg_ in ascites showed increased expression of PD-1 (42.45 ± 8.02% of T_reg_) and PD-L1 (30.75 ± 10.84% of T_reg_), whereas the proportions of tumor-infiltrated PD-1+ T_reg_ and PD-L1+ T_reg_ were 18.30 ± 5.64% and 36.57 ± 15.48%, respectively (**Figure 4H**). The investigated immune checkpoint markers were almost absent on blood-circulating T_reg_ with < 10% marker expression on average.

Moreover, CD4+ and CD8+ αβT cells were classified according to their differentiation stages: T_N_ (naïve T cells: CD45RA+ CCR7+ CD28+ CD95-), T_SCM_ (stem cell-like memory T cells: CD45RA+ CCR7+ CD28+ CD95+), T_CM_ (central memory T cells: CD45RA- CCR7+ CD28+), T_TM_ (transitional memory T cells: CD45RA- CCR7- CD28+), T_EM_ (effector memory T cells: CD45RA- CCR7- CD28-), and T_TE_ (terminal effector T cells: CD45RA+ CCR7- CD28-). In general, the differentiation profiles of CD4+ and CD8+ T cells in the three investigated OC-associated compartments were very similar. For both CD4+ and CD8+ T cells, significantly elevated T_N_ proportions were found in PB samples, with 29.31 ± 2.97% of CD4+ T cells and 15.07 ± 2.57% of CD8+ T cells, compared to the other two compartments (**Figures 4I, J**). The predominance of less differentiated blood-circulating CD4+ and CD8+ T cells was also visible for T_SCM_ and T_CM_, although not reaching a significant difference compared to the other two OC-associated compartments. Further, CD4+ T_TM_ dominated in ascites and tumor tissues with 53.64 ± 10.37% and 46.63 ± 8.79% of CD4+ T cells, respectively. Likewise, CD8+ T_TM_ and CD8+ T_EM_ proportions were the most abundant differentiation stages in ascites (T_TM_: 27.42 ± 6.06% of CD8+ T cells, T_EM_: 21.11 ± 6.46% of CD8+ T cells) and tumor samples (T_TM_: 28.62 ± 5.97% of CD8+ T cells, T_EM_: 38.84 ± 7.75% of CD8+ T cells). In comparison to PB of HD, OC patients showed significantly reduced frequencies of CD4+ and CD8+ T_N_ as well as higher amounts of CD4+ and CD8+ T_SCM_ (**Supplementary Figure 11**).

Thus, comprehensive analysis of adaptive immune cells in multiple compartments of high-grade serous OC patients showed that the proportions of tumor-infiltrated CD3+ T cells, especially CD4+ T cell subsets, were diminished compared to both liquid samples. However, higher proportions of potentially immunosuppressive T_reg_ were found in OC tissues than in ascites and PB samples. Further, blood-circulating αβT cells predominantly displayed less differentiated phenotypes (T_N,_ T_SCM_), whereas in the other two compartments γδT cells and αβT cells mainly possess memory differentiation stages (γδT_CM_, γδT_EM_, αβT_TM_, αβT_EM_) representing T cells with increased effector functions and cytotoxicity.

### 3.3 Profiling of activating and inhibitory receptor expression by αβ T cells

**Figure 5:**
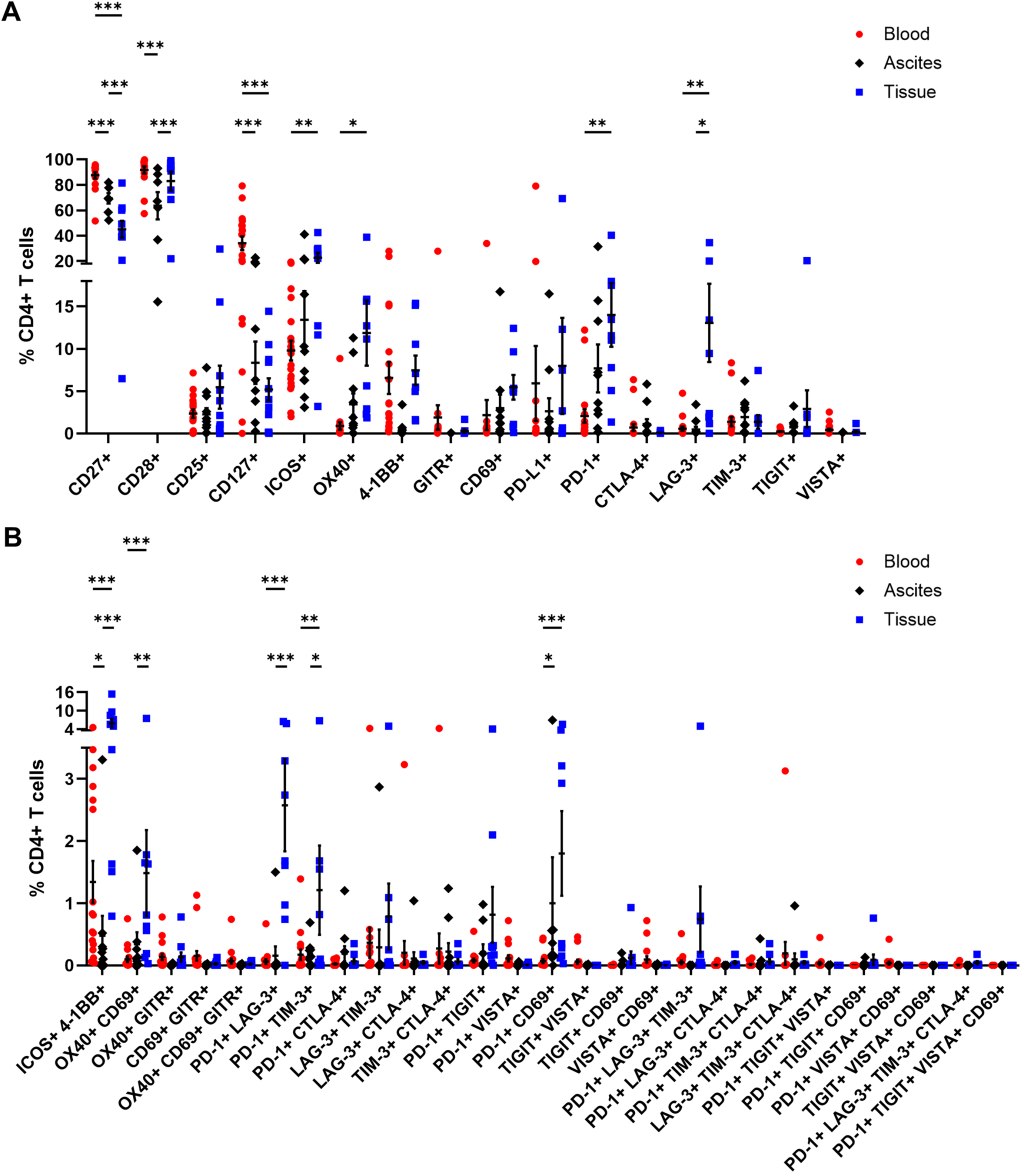
Receptor expression profiling of CD4+ T cells in matched peripheral blood, ascites, and tumor tissue samples. **(A)** Dot plots show proportions of CD4+ T cells expressing single co-activation and co-inhibitory markers. **(B)** Proportions of CD4+ T cells expressing two to four receptor combinations calculated by Boolean gating in FlowLogic™ using AND function are shown. Mean ± SEM, two-way ANOVA, Bonferroni *post-hoc* test, *p ≤ 0.05, **p ≤ 0.01, ***p < 0.001.

**Figure 6:**
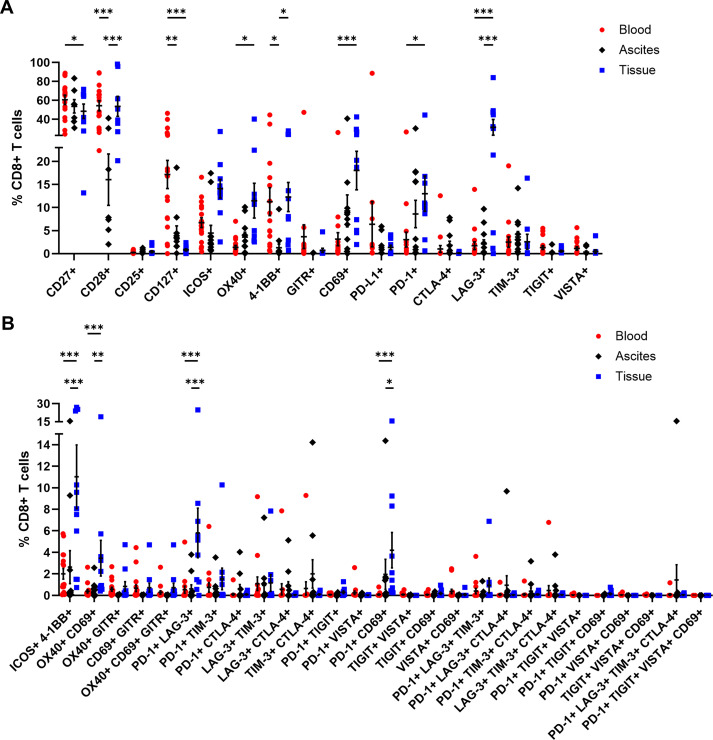
Receptor expression profiling of CD8+ T cells in matched peripheral blood, ascites, and tumor tissue samples. **(A)** Dot plot show proportions of CD8+ T cells expressing single co-activation and co-inhibitory markers. **(B)** Proportions of CD8+ T cells expressing two to four receptor combinations calculated by Boolean gating in FlowLogic™ using AND function are shown. Mean ± SEM, two-way ANOVA, Bonferroni *post-hoc* test, *p ≤ 0.05, **p ≤ 0.01, ***p < 0.001.

In addition, CD4+ and CD8+ T cells were comprehensively analyzed in terms of their expression of several activating or inhibitory marker proteins (53–55). In general, the proportions of all investigated receptors in all three studied OC-associated compartments were similar for CD4+ and CD8+ T cells (**Figures 5**, **6**). Starting with T cell stimulating receptors, percentages of CD27 expression were highest on blood-circulating CD4+ T cells (87.52 ± 2.57% of CD4+ T cells) and CD8+ T cells (60.59 ± 4.98% of CD8+ T cells) followed by ascites samples and tumor tissues having least CD27+ T cell proportions (**Figures 5A**, **6A**). This graduation in T cell proportions was also visible for CD127 (interleukin-7 receptor, IL-7R) expression, with PB having significantly elevated CD127+ T cell proportions (34.05 ± 5.40% of CD4+ T cells and 17.16 ± 3.05% of CD8+ T cells). Moreover, ascites-derived CD4+ T cells and CD8+ T cells exhibited significantly reduced proportions of CD28, with 63.60 ± 10.77% of CD4+ T cells and 16.05 ± 5.57% of CD8+ T cells, compared to PB and tumor samples. Considering the percentages of the other activating receptors ICOS, OX40, and 4-1BB by CD4+ T cells and CD8+ T cells, highest proportions were found in tumor-infiltrated T cells. In addition, proportions of ICOS+ 4-1BB+ T cells were significantly increased in tumor tissues being at least 3-times more abundant compared to both liquid samples (**Figures 5B**, **6B**). In addition, CD8+ T cells demonstrated significantly higher proportions expressing the activation marker CD69+ in the tumor tissues (18.03 ± 4.17% of CD8+ T cells) compared to PB (3.14 ± 1.43% of CD8+ T cells). In terms of detailed inhibitory receptor expression analysis, significantly higher fractions of tumor-infiltrated CD4+ T cells expressed LAG-3 (13.06 ± 4.63% of CD4+ T cells) and PD-1 (14.01 ± 3.76% of CD4+ T cells, **Figure 5A**). Similarly, significantly elevated percentages of CD8+ T cells expressing LAG-3 (31.50 ± 8.33% of CD8+ T cells), and PD-1 (13.01 ± 3.82% of CD8+ T cells) were detected in tumor tissues (**Figure 6A**), as well. In comparison, lower proportions of ascites-derived CD4+ T cells and CD8+ T cells expressed PD-1 (7.69 ± 2.84% of CD4+ T cells, 8.60 ± 2.96% of CD8+ T cells). Looking at T cells in the PB, inhibitory marker expression was almost absent with < 8% of CD4+ or CD8+ T cells. Furthermore, co-expression of PD-1 and LAG-3 or PD-1 and CD69 were the two most common marker combinations on T cells in OC tissues (**Figures 5B**, **6B**).

Generally, tumor-infiltrated CD4+ and CD8+ T cells displayed higher proportions expressing one or multiple activating and inhibitory markers than T cells in the liquid compartments (**Supplementary Figure 12**). Comparing PB of OC with HD, we found significant higher proportions of CD4+ T cells expressing CD25, CD127, or 4-1BB as well as CD8+ T cells positive for CD25, CD127, ICOS, OX40, or 4-1BB in the blood of OC patients compared to HD (**Supplementary Figures 13**, **14**).

### 3.4 Association of immune cell proportions and phenotypes with clinicopathological patient characteristics and clinical outcome

In order to associate the immune cell profiles with clinicopathological OC patient characteristics, like FIGO stage, serum CA125 level at surgery, or residual tumor after surgery, the previous main cohort of 24 patients was expanded by four additional OC patients, where only PB samples were received for immune cell monitoring. Regarding immune cell abundancies and phenotypic marker expression, the results of the four additional patients did not differ significantly from the previous PB samples (**Supplementary Figure 15**).

Immune cell phenotypes within the three investigated OC-associated compartments were compared between patients classified as FIGO stage III and FIGO stage IV (**Supplementary Figure 16**). In our cohort, one third of the patients were classified as FIGO stage IV (**Table 1**). Thereby, patients with FIGO stage IV exhibited significantly reduced CD8+ T cell proportions in tumor tissues (FIGO III: 30.98 ± 5.16% of CD3+ T cells, FIGO IV: 16.33 ± 3.70% of CD3+ T cells) being visible as trend also for ascites and PB samples (**Supplementary Figure 16B**). In PB samples of patients with FIGO stage IV, percentages of cDC2, CD57+ CD16- NK cells, and CD4+ T_TM_ were increased, whereby these differences were not seen in ascites or tumor samples. In addition, CA125 was measured in patient serum prior to surgery, as CA125 is the best-characterized biomarker in OC so far (40) and elevated serum CA125 level were linked to unfavorable clinical outcomes in OC patients (10, 11). We investigated, whether immune cell frequencies and phenotypes can be associated with serum CA125 level (**Supplementary Figure 17**). The average serum CA125 value at surgery for our cohort was 487.5 IU/ml. In PB samples of OC patients with high CA125 values (> 487.5 IU/ml), the proportions of CD40+ pDC and TIGIT+ CD8+ T cells were significantly raised, with ascites and tumor tissue samples showing the same trend for both immune cell phenotypes (**Supplementary Figure 17B**). Patients with high CA125 level had also increased percentages of ICOS+ CD4+ T cell in ascites, with the other two compartments indicating same trends for both immune cell subtypes. In addition, patients were stratified according to their surgical outcome and immune cell frequencies were compared between patients with complete resection and residual tumor, whereby almost 80% of our patients underwent macroscopic complete resection (**Table 1**, **Supplementary Figure 18**). OC patients with residual tumor after surgery demonstrated elevated levels of PB-circulating PD-L1+ non-classical monocytes as well as tumor-infiltrated neutrophils, e-MDSC, γδT_CM_, and PD-L1+ γδT cells, with the corresponding other OC-associated compartments showing the same trends for these immune cell phenotypes (**Supplementary Figure 18B**). On the contrary, γδT_EM_ cells were more pronounced in ascites and tumor samples of patients with complete resection.

**Figure 7:**
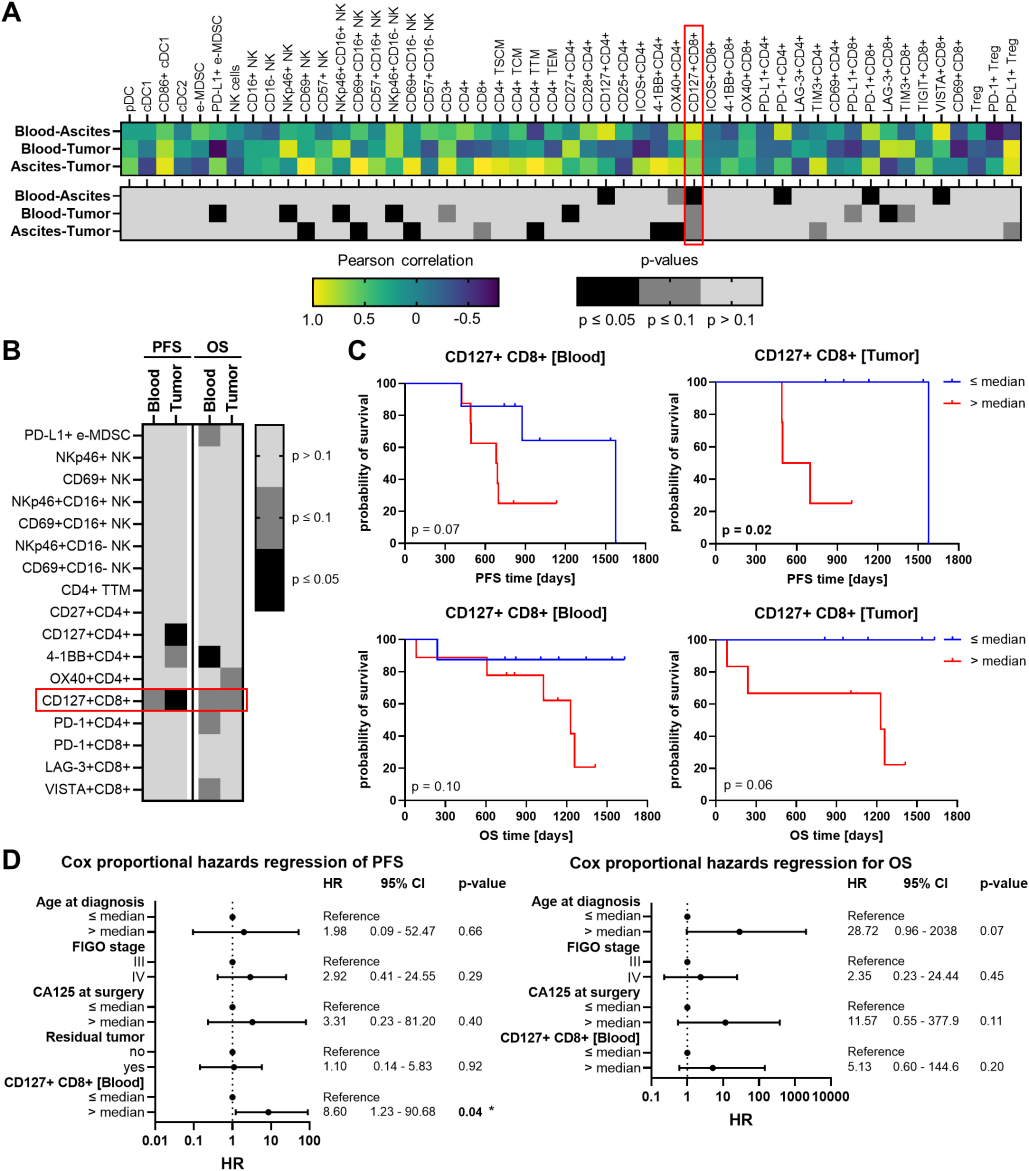
Correlation analysis of investigated immune cells in matched peripheral blood, ascites, and tumor tissue samples and their association with high-grade serous OC patients’ survival. **(A)** Heatmaps show Pearson correlation coefficients (r, top) and corresponding p-values (two-tailed, bottom) of correlations between the frequency of each individual immune cell subsets in peripheral blood (PB) and ascites, PB and tumor tissues, or ascites and tumor tissues. Values are depicted only for immune cell markers with matched pairs in all three compartments. **(B)** The heatmap-like graph shows p-values calculated by Log-rank test of progression-free (PFS) and overall survival (OS), where OC patients were stratified by respective median immune cell frequencies in PB and tumor and **(C)** separately presented Kaplan-Meier curves for PFS and OS of OC patients stratified by median CD127+ CD8+ T cell frequencies in PB and tumor. **(D)** Cox proportional hazards regression models for PFS (left) and OS (right) entailing age at diagnosis (median = 69 years), FIGO stage (III or IV), CA125 at surgery (median = 487.5 IU/ml), residual tumor after surgery (no – macroscopically complete resection, yes – incomplete resection), and frequency of CD127+ CD8+ T cells in peripheral blood (median = 15.07% of CD8+ T cells). Hazard ratio (HR) with 95% confidence interval (CI) and p-value.

In order to find a potential prognostic biomarker reflecting the local TME in one of the liquid biopsies, the immune cell characteristics in the OC-associated compartments were correlated with each other resulting in three comparisons (blood - ascites, blood - tumor, ascites - tumor). Therefore, we included only matched pairs of all three regions and excluded immune cell subsets with very low abundancies. For each immune cell phenotype, calculated Pearson correlation coefficients and corresponding p-values for the three comparisons are shown in separate heatmap-like graphs (**Figure 7A**). Generally, the correlation analysis showed that the three comparisons resulted in similar numbers of significant correlations. Looking at individual correlations of immune cell phenotypes among the OC-associated sites in more detail, CD127+ and PD-1+ T cell subpopulations correlated significantly positive between both liquid samples. In particular, CD127+ CD8+ T cells demonstrated significant or trending positive correlations between all three investigated compartments. Further, CD27+ CD4+ T cells and LAG-3+ CD8+ T cells displayed significant positive correlations between PB and tumor tissues, whereas a positive association of 4-1BB+ CD4+ or OX40+ CD4+ T cells derived from ascites and tumor samples was detected. Regarding NK cells, significantly positive correlations were obvious for NKp46+ NK cells and CD69+ NK cells in PB or ascites samples in association with corresponding tumor tissues, irrespective of CD16 expression.

Further, immune cell populations showing significant correlations among the investigated compartments were associated with progression-free survival (PFS) and OS of OC patients. In this regard, only patients with a follow up period of at least two years were included (**Table 1**, **Supplementary Figure 19**). Due to the limited number of ascites samples from patients with suitable clinical data, only immune cells in PB and tumor tissue were appropriate for survival analysis. OC patients were stratified according to the median abundance of the respective immune cell subset and calculated p-values with respect to PFS and OS are shown in a heatmap-like manner (**Figure 7B**). OC patients with high frequencies of either tumor-infiltrating CD127+ CD4+ T cells or blood-circulating 4-1BB+ CD4+ T cells showed significantly reduced PFS and OS, respectively (**Supplementary Figure 20**). However, CD127+ CD8+ T cells were the only immune cell subset in both, PB and tumor tissues, clearly associated with PFS and OS, with high CD127+ CD8+ T cell proportions being linked to unfavorable OC patients’ outcomes (**Figure 7C**). Further, multivariate Cox regression was performed to investigate whether proportions of CD127+ CD8+ T cells in PB might be an independent prognostic marker. Therefore, patient age at diagnosis, FIGO stage, serum CA125 at surgery, and residual tumor after surgery were included in the Cox proportional hazards regression models (**Figure 7D**). Of note, surgical outcome was only integrated for PFS and not for OS regression model due to missing OS events within the two-year follow-up period of patients with residual tumor. For both OS and PFS of our OC cohort, higher age at diagnosis (> 69 years), higher FIGO stage (IV), and higher CA125 values (> 487.5 IU/ml) were linked to increased hazard ratios (HR). The association with poor prognosis was also evident for CD127+ CD8+ T cell abundance, with a high proportion (> 15% of blood-circulating CD8+ T cells) being an independent prognostic marker for unfavorable PFS (HR = 8.61, p = 0.04).

### 3.5 Characterization of CD127+ CD8+ T cells

**Figure 8:**
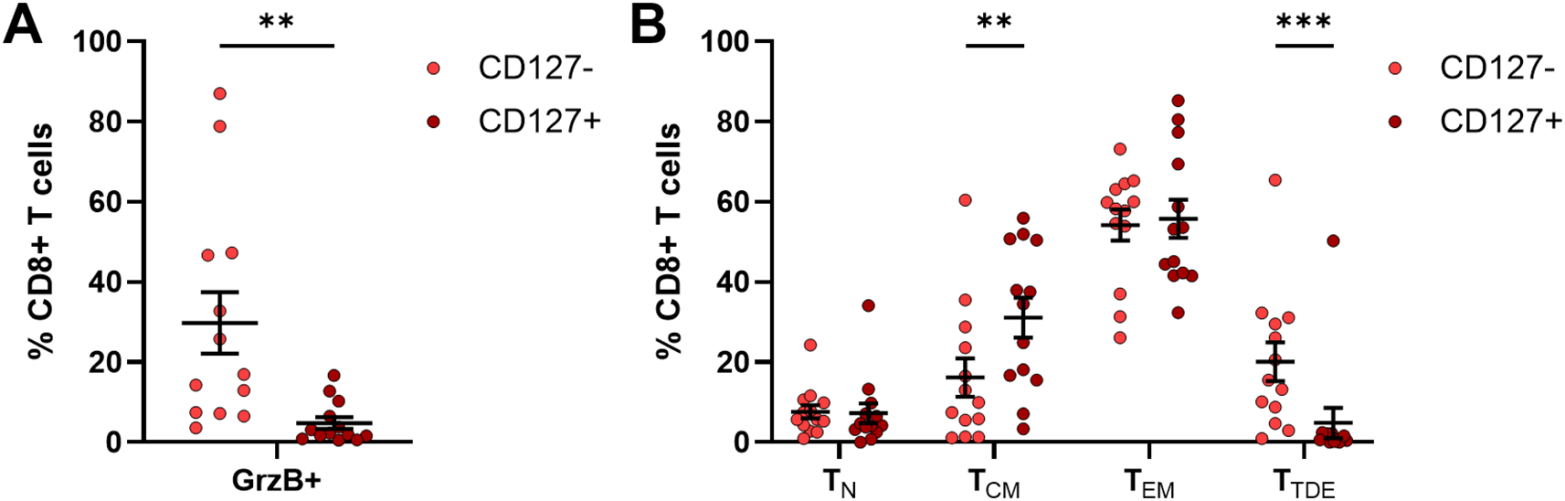
Granzyme B production of CD127+ CD8+ T cells from frozen PBMC samples of OC patients. Flow cytometric analysis of 13 frozen PBMC samples isolated from peripheral blood samples with respect to **(A)** granzyme B (GrzB) expression of CD127+ CD8+ T cells in comparison to CD127- CD8+ T cells and **(B)** maturation stages of CD127**+** CD8+ T cells and their CD127- counterparts after 4h incubation with PMA, ionomycin, and brefeldin A Mean ± SEM, paired t-test, **p ≤ 0.01, ***p < 0.001.

In addition to the comprehensive investigation of immune cells freshly isolated from PB samples, remaining frozen PBMC samples from 13 OC patients were used to assess the expression of GrzB and maturation status of CD127+ CD8+ T cells compared to their CD127- counterparts by flow cytometry after stimulation. A significantly reduced GrzB expression by CD127+ CD8+ T cells was detected being six-times lower than in CD127- CD8+ T cells (**Figure 8A**). Comparing the frequency of GrzB+ CD127+ CD8+ T cells in OC patient samples with PB of HD, GrzB expression by CD127+ CD8+ T cells was significantly lower in OC (**Supplementary Figure 21A**).

Beyond a reduced cytotoxic potential, the proportion of terminally differentiated effector CD127+ CD8+ T cells (T_TDE_: 4.86 ± 3.80% of CD8+ T cells) in OC, defined as CD45RA+ CCR7- T cell population, was significantly lower than corresponding CD127- CD8+ T cells (T_TDE_: 20.10 ± 4.84% of CD8+ T cells, **Figure 8B**). CD127+ CD8+ T cells showed predominantly memory phenotypes (T_CM_: CD45RA- CCR7+, T_EM_: CD45RA- CCR7-), whereby a significantly higher proportion of central memory CD127+ CD8+ T cells was measured (CD127+ CD8+ T_CM_: 31.14 ± 5.01% of CD8+ T cells, CD127- CD8+ T_CM_: 16.20 ± 4.78% of CD8+ T cells). In comparison to HD, OC patients showed significantly reduced proportions of CD127+ CD8+ T_N_ and CD127- CD8+ T_N_ (**Supplementary Figure 21B**), but increased frequencies of CD127+ CD8+ T_EM_ and CD127- CD8+ T_EM_. Thus, the characterization of stimulated CD127+ CD8+ T cells revealed a memory T cell phenotype with reduced potential to produce GrzB in OC, explaining why an increased CD127+ CD8+ T cell population might contribute to an unfavorable prognosis of OC patient.

Taken together, the proportion of cytotoxic CD8+ T cells decreased in advanced stage OC (FIGO stage IV), especially in tumor tissues. Further, frequencies of CD127+ CD8+ T cells correlated positive between liquid samples and tumor tissues, with high proportions in PB and tumor tissue being associated with shorter PFS and OS of high-grade serous OC patients. Thereby, high proportions of blood-circulating CD127+ CD8+ T cells, as effector memory T cells with low cytotoxic activity, turned out to be an independent prognostic marker for a higher risk of tumor progression.

## 4 Discussion

OC is a highly heterogeneous tumor entity with tumor progression and metastasis being accompanied by ascitic fluid accumulation in the peritoneal cavity, especially in advanced tumors. Due to the lack of early detection and effective long-lasting treatment options, advanced disease stages are associated with high mortality (56–58). In this context, immune cells might influence survival of OC patients and responses to different therapeutic strategies (59–62). In our study, we comprehensively investigated matched samples from PB, ascites, and tumor tissues of high-grade serous OC patients with regard to infiltration, differentiation, and phenotype of various immune cells.

DC play a pivotal role in anti-tumor immunity by initiating and controlling innate as well as adaptive immune responses (63). pDC and cDC comprised less than 6% of HLA-DR+ cells in the three investigated compartments of our OC cohort. However, highest DC frequencies, especially cDC2 proportions, were detected in ascites confirming single-cell RNA sequencing experiments (64, 65). Our results are also in line with a study by Pawłowska et al. showing significantly increased myeloid DC and pDC frequencies in peritoneal fluid compared to PB and tumor tissues of patients with early and advanced OC (44). In contrast, Vazquez et al. reported a power of ten higher pDC frequencies in ascites samples compared to our study (10). This discrepancy in terms of pDC abundance might be due to different detection markers used in flow cytometric analysis. Vazquez et al. utilized CD123 as pDC marker and calculated pDC proportions of HLA-DR+CD16-CD14- cells, whereas we defined pDC as CD123+ CD303+ cells among HLA-DR+ cells. Further, pDC are supposed to set up an immunosuppressive microenvironment in OC through ICOS-L expression and the activation of ICOS+ T_reg_ (66, 67). Our data support this, as tumor-infiltrated as well as ascites-derived pDC showed increased proportions of ICOS-L+ cells for a potential T_reg_ interaction. Looking at the DC phenotypes in more detail, pDC and cDC1 in tumor tissues and to a lesser extent in liquid samples demonstrated a more mature phenotype based on significantly higher proportions positive for CD86 and CD40. Previously published data analyzing blood samples showed no differences in CD86+ cDC proportions during therapy of OC patients (68), while others detected higher proportions of CD86+ cDC1 in comparison to CD86+ cDC2 (26).

Furthermore, classical monocytes were the prevalent subtype in PB with about 70% of CD14+ cells, while < 10% of CD14+ cells were classified as intermediate and non-classical monocytes. This is partially in line with a study by Prat et al. observing about 60% classical and 35% intermediate monocytes in blood samples of OC patients (69). Further, increased percentages of intermediate monocytes were found in ascites indicating a transitional state of ascites-derived monocytes from classical to non-classical monocytes (70).

In our multi-compartment analysis, neutrophils isolated from ascites showed a mature phenotype (CD16+ CD10-). In contrast, a more immature phenotype (CD16- CD10-) was assigned to tumor-infiltrated neutrophils in our OC patient cohort. Comparing PB neutrophils of OC patients with healthy controls, Rice et al. described higher percentages of immature neutrophils in OC blood samples due to reduced CD10 and CD16 expression (71). Further, the exposition of donor neutrophils with ascitic fluid resulted in an increased CD10 expression reflecting an induced neutrophil maturation (72, 73). Besides OC, immature neutrophils within the tumor microenvironment have also been described for lung and breast cancer patients. However, immature as well as mature neutrophils can have immunosuppressive effects (46). In addition, in our cohort, a high proportion of ascites-associated neutrophils exhibited MPO expression being associated with neutrophil extracellular traps (NETs) (74). As an early response towards the presence of OC cells in the abdominal cavity, neutrophils form and release NETs trapping the cancer cells and leading to the formation of metastasis on the omentum and, thus, contributing to tumor progression (75).

In OC patients, high abundance of immunosuppressive MDSC in PB, ascites or tumor biopsies correlated with metastasis and lower survival probabilities (21, 76). Our analysis of the different MDSC subpopulations revealed significantly increased tumor infiltration by PMN-MDSC in comparison to the liquid biopsies. This finding is partially in line with a recent study by Okła et al. investigating a cohort of epithelial OC patients and reporting significantly elevated PMN-MDSC and M-MDSC proportions in tissues (41). Furthermore, in advanced (FIGO stage III-IV) and high-grade (G3) OC, the authors found also elevated proportions of e-MDSC in PB samples compared to ascites and tumor. This data supports the idea that tumor cells in OC patients create a highly immunosuppressive microenvironment in the tumor.

In our analysis, NK cell proportions were comparable among all investigated OC-associated compartments that is in line with Tonetti et al. (77), whereas other studies demonstrated higher NK cell percentages in ascites samples compared to PB (11, 78). However, tumor-infiltrated NK cells exhibited significantly reduced proportions of CD16+ subpopulations in comparison to NK cells isolated from liquid samples. Thereby, we confirm other investigators describing immature NK cells in OC that also have reduced anti-tumoral activity (6, 7, 31, 78–81). Impaired functionality of tumor-infiltrated NK cells was confirmed in our experiments due to the absence of activating receptors (NKG2D, NKp46) and the differentiation marker CD57. Similarly, Henriksen et al. found low densities of CD57+ NK cells in OC tumor tissues by immunohistochemical staining (82). Within the TME, the persistent and excessive ligand exposure might result in the downregulation of activating receptors and impairment of NK cell activity (77, 83, 84). However, a significantly higher proportion of NKp46+ NK cells was detected in ascites compared to the other two investigated compartments. This is in line with Kumar et al. showing a significant increase of NKp46+ CD56dim NK cells in peritoneal fluid (17). Furthermore, significant positive correlations between blood-circulating and tumor-infiltrated NKp46+ NK cells were found in our cohort, while CD69+ NK cells correlated significantly positive between ascites and tumor. Besides NK cell activation, CD69 expression on NK cells might also indicate tissue residency (85, 86). In this context, CD69+ NK cells potentially represent a fraction of tissue-resident NK cells, especially within the TME and in tumor-associated compartments, like ascites. Thus, there might be different NK cell-mediated anti-tumoral responses in the analyzed compartments representing the unique microenvironments.

γδT cells are a small T cell subset being activated in a T cell receptor (TCR)-dependent or TCR-independent manner (50). Regarding the abundance of γδT cells, several studies demonstrated relatively low proportions of ascites- (3.4 – 3.7% of T cells) and tumor-derived γδT cells (1.5 – 1.8% of T cells) among OC patients (11, 50), being confirmed by our results. Rådestad et al. found CD4- CD8- γδT cells to be the predominant γδT cell phenotype, followed by CD8+ γδT cells, and CD4+ γδT cells, which were significantly increased in tumor tissues compared to ascites samples (11). In our study, CD4+ γδT cells represented the most frequent γδT cell subtype, especially in OC tissues. Moreover, memory subsets represented the predominant differentiation stages of γδT cells, with γδT_CM_ (CD27+) being significantly increased in tumor tissue and γδT_EM_ (CD27-) dominating in ascites. In this context, higher proportions of CD27low/- γδT cells were found in ascites compared to tumor tissue samples of OC patients (50) being confirmed by our results. Tumor-infiltrated γδT cells showed significantly increased proportions expressing CD69 and PD-L1. As γδT cells upregulate CD69 and PD-1 during activation, whereby PD-1 expression triggers inhibitory signals within the γδT cell itself, γδT cells seemed to be active in OC tissues and might inhibit effector αβT cells via PD-L1 – PD-1 axis (87–89).

In terms of αβT lymphocyte abundance, proportions of tumor-infiltrated CD3+ and CD4+ T cells were significantly lower than in the two liquid compartments. The findings were in line with other reported studies (11, 64, 77, 90). On the contrary, CD8+ T cell proportions were the highest in ascites samples confirming single-cell RNA sequencing results by Zheng et al., who investigated PB, primary ovarian tumor, matched omentum metastasis, and ascites from OC patients (64). In addition, patients with OC classified as FIGO stage IV exhibited significantly lower proportions of cytotoxic CD8+ T cells compared to FIGO stage III samples. These data clearly reflect the progressive immune evasion at higher FIGO stages. Similarly, Rådestad et al. found increased percentages of total CD3+ T cells, but no significant changes in CD8+ T cell subsets in ascites and tumor tissue samples of OC patients with FIGO stage IV compared to FIGO stage III (11). We also analyzed T_reg_ that were most abundant in tumor tissues compared to ascites and PB samples. This graduation is supported by studies investigating ascites, primary tumors, or metastases of OC patients (11, 64, 91). We also found that ascites- and tumor tissue-derived T_reg_ exhibited high expression of immunosuppressive PD-1 and PD-L1 that is proven by the literature (92).

Looking at the differentiation status of CD4+ and CD8+ T cell populations, high frequencies of T_TM_ and T_EM_ subpopulations, representing T cells with increased effector function and cytotoxicity, were particularly noticeable in ascites and tumor samples. In contrast, T_N_ and T_SCM_ were most abundant in PB samples indicating different TME in the investigated compartments that induced diverse T cell differentiation patterns. We distinguished six T cell differentiation stages using CD28, CD95, CD45RA, and CCR7. Nevertheless, we confirmed a single-cell RNA sequencing study by Zheng et al. (64) and flow cytometry experiments by other investigators that detected higher levels of T_N_ (CD45RA+ CCR7+) and T_EM_ (CD45RA− CCR7−) in blood and ascites or tumor tissues (11, 36, 90, 93). Further, in our OC cohort, the analysis of activating and inhibitory receptors on T cells showed that tumor-infiltrated T cells most frequently expressed one or more receptors, followed by ascites-derived T cells and PB-circulating T cells. Looking at the expressed receptors in more detail, higher proportions of CD27+, CD28+, OX40+, ICOS+ and/or 4-1BB+ T cells as well as PD-1+ and/or LAG-3+ or CD69+ T cells were detected being in line with Rådestad et al. (11). These authors also found elevated levels of tumor-infiltrated TIM-3+ T cells, which we could not confirm. Moreover, Tassi et al. identified a special T cell subtype (CD137+ CD39+ PD-1+ TIM-3+ CD45RA- CD62L- CD95+) in OC tissues and not ascites or PB of patients (94). In literature, a pronounced immunosuppressive TME of primary OC and OC metastases in comparison to ascitic fluid was reported referring to elevated PD-1+ CD8+ T cell frequencies in tumor samples (64). However, in a cohort of chemotherapy-naïve high-grade serous carcinoma patients, tumor-infiltrated T cells expressing PD-1, LAG-3, and CTLA-4 were linked to favorable relapse-free survival and OS, whereas TIM-3+ cells were associated with reduced OS (95). James et al. described both ICOS and LAG-3 to positively associate with favorable survival (38). The expression of multiple inhibitory receptors, especially PD-1, LAG-3, CTLA-4, and TIM-3 by T cells probably indicate exhausted and dysfunctional T cell phenotypes (11, 95–99). However, activated effector T cells express these receptors as well (100–102) and intratumoral T cells having a terminally differentiated maturation phenotype might also express LAG-3 (103). In our study, tumor-infiltrated T cell populations most probably represented a mixture of activated T cells, effector memory T cells, and potentially exhausted T cells, due to either CD45RA- CCR7- (T_TM_/T_EM_) or CD27+, CD28+, ICOS+ and/or 4-1BB+, OX40+, PD-1+ and/or LAG-3+ or CD69+ phenotypes lacking TIM-3 and CTLA-4 expression. Further, tumor-infiltrated T cells showed a significant downregulation of CD127, which is a sign of active T cells as effector T lymphocytes downregulate IL-7R expression after activation (11, 104). The significantly lower expression of CD27 and CD127 by tumor-infiltrated T cells in our cohort might emphasize their effector memory state (104, 105). In particular, the frequency of CD127+ CD8+ T cells correlated significantly positive among the matched PB, ascites, and tumor tissue samples of our cohort. Patients with high abundancies of CD127+ CD8+ T cells in blood and tumor tissues, reflecting not activated effector T cells, showed reduced PFS and OS. Similarly, Rådestad et al. found an association of increased TIM-3-expressing CD127+ CD8+ T cell proportions with diminished OS of OC patients (11). Moreover, lower frequencies of PB-circulating CD127+ lymphocytes (< 5%) were associated with beneficial survival in rectal and breast cancer patients, but not in patients diagnosed with liver, gastric, esophageal, ovarian, or colon cancer (106). However, a higher frequency of blood-circulating CD127+ CD8+ T cells (> 15% of CD8+ T cells) turned out to be an independent prognostic biomarker for unfavorable PFS in our OC patient cohort (n = 24-28). Nevertheless, the biomarker potential of CD127+ CD8+ T cell frequencies should be further validated in larger patient cohorts.

## 5 Conclusion

The comprehensive analysis of innate and adaptive immune cells showed that the general abundance of immune cells was comparable between matched PB, ascites, and tumor tissues. However, differentiation and phenotypic marker expression varied significantly among the investigated immune cell subsets. In PB, mature NK cells and less differentiated T cells were increased, while DC and mature neutrophils were prevalently found in ascites. In OC tissues, higher proportions of immature neutrophils, CD16- NK cells, and effector memory T cells were observed, although the percentages of intratumoral T cells were significantly reduced compared to the other two compartments. Especially, patients with FIGO stage IV exhibited markedly lower CD8+ T cell abundancies. In addition, high proportions of T cells expressing one or multiple activating and inhibitory receptors in tumor tissues emphasized the presence of effector memory, activated, and exhausted T cells. In particular, significant positive correlations of CD127+ CD8+ T cells among the studied OC-associated sites underlined their predictive potential of OC patient stratification in terms of clinical outcome. CD127+ CD8+ T cells showed a low GrzB production and the majority belonged to memory T cell phenotypes in PB of OC patients. Thereby, we identified high CD127+ CD8+ T cell frequencies in PB (> 15% of blood-circulating CD8+ T cells) as an independent prognostic marker for a higher risk of tumor progression in high-grade OC patients.

## Data Availability

The raw data supporting the conclusions of this article will be made available by the authors, without undue reservation.
