# Supplementary material for: Multi-compartment immune cell profiling highlights the prognostic relevance of CD127+ CD8+ T cells for patients with high-grade serous ovarian cancer
Source: Front Immunol. 2025 Oct 27;16:1607471. doi: 10.3389/fimmu.2025.1607471 (PMC12597928; doi:10.3389/fimmu.2025.1607471)
Supplement: Supplementary file 1 [file DataSheet1.docx]

Supplementary Material

# Supplementary Figures

**
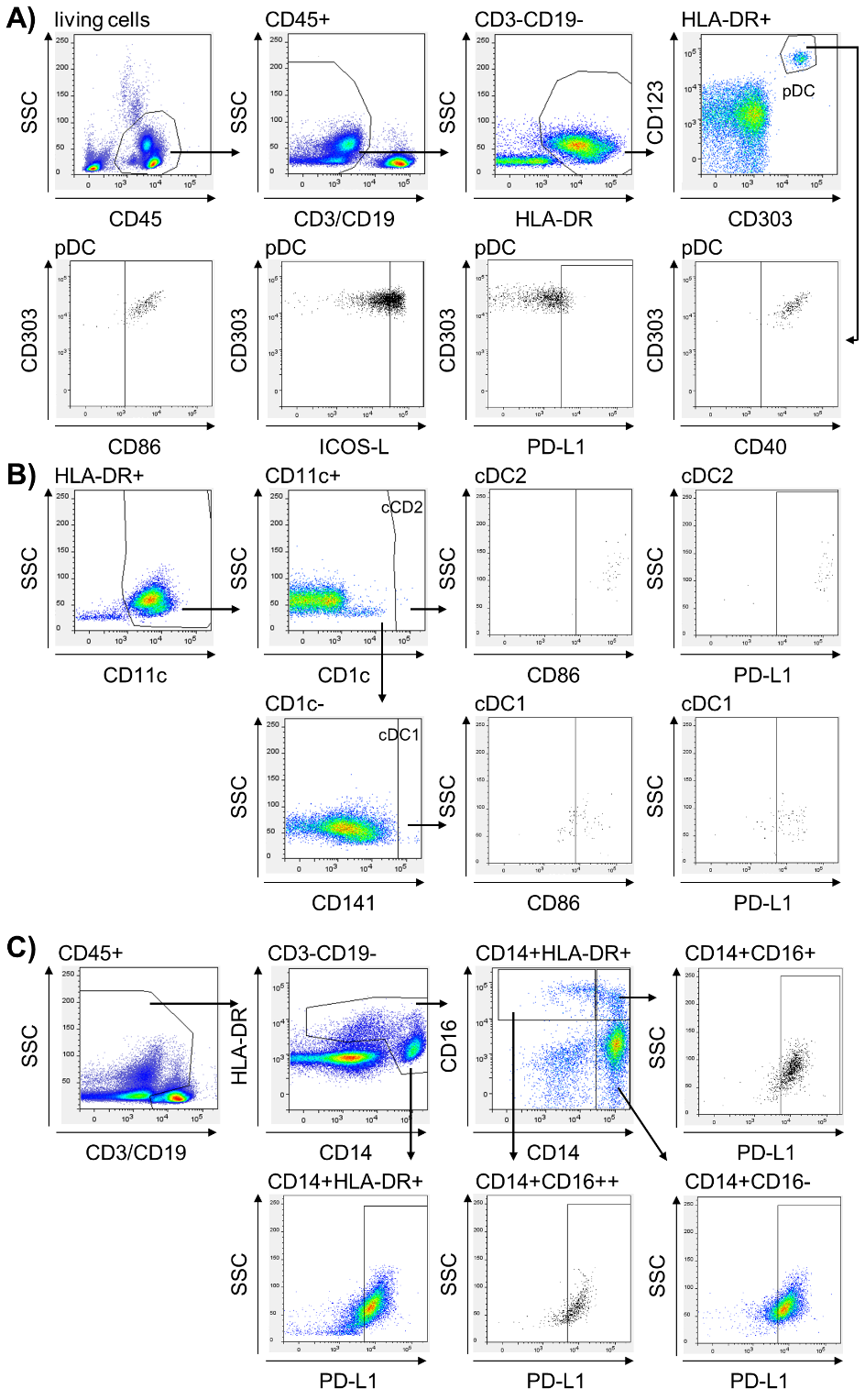
**

**Supplementary Figure 1.** **Exemplary gating strategies of DC and monocyte subpopulations.** Gating strategies of **A**) pDC and **B)** cDC (cDC1: CD141+, cDC2: CD1c+) with their respective expression pattern, e.g. for CD86 or PD-L1, as well as **C)** monocytes according to their CD14 and CD16 or PD-L1 expression are exemplarily shown on blood (A [top], B), tumor tissue (A [CD86, CD40]), or ascites (A [ICOS-L, PD-L1]) samples.


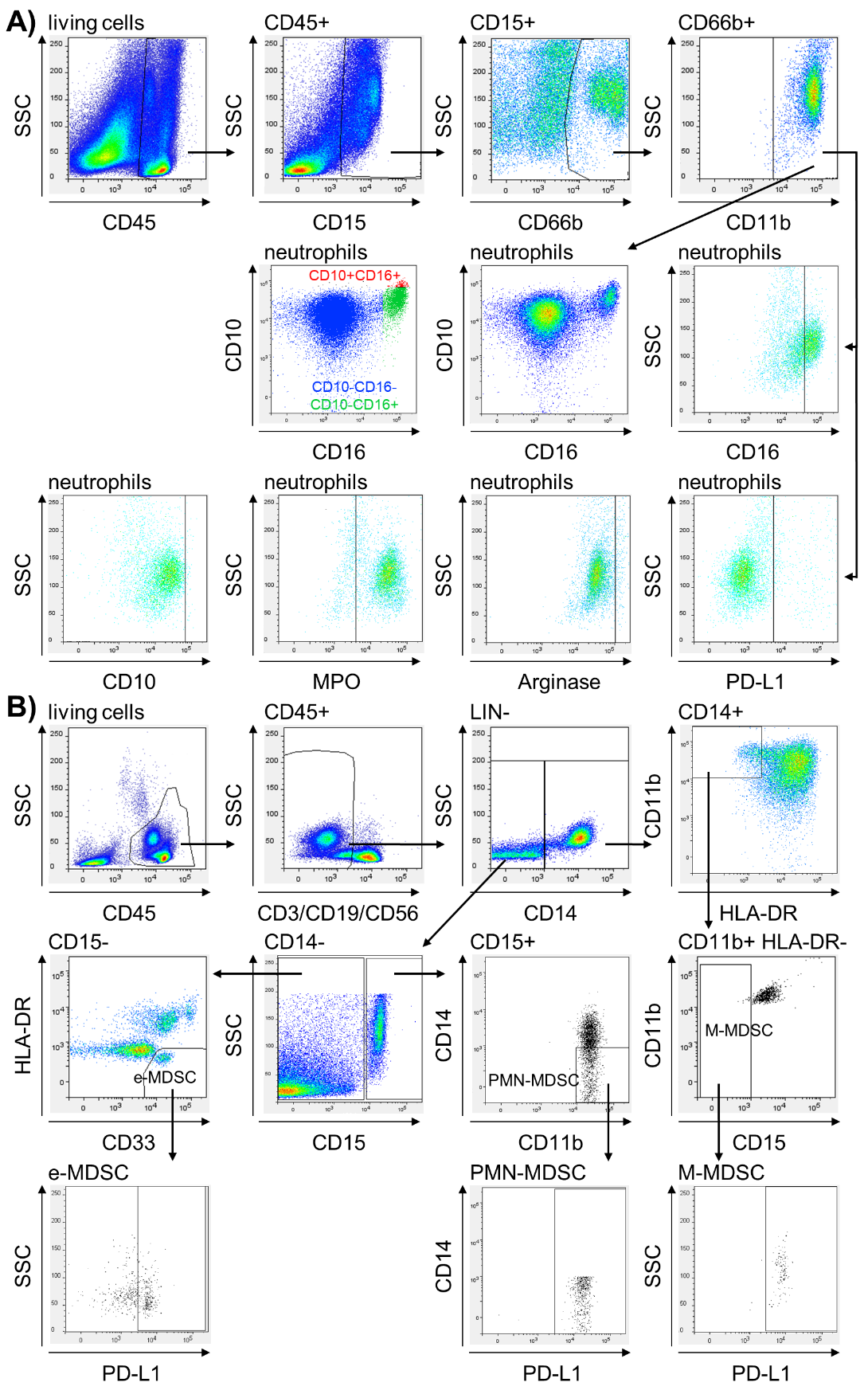


**Supplementary Figure 2.** **Exemplary gating strategies of neutrophils and MDSC.** Gating strategies of **A)** neutrophils (CD15+ CD66b+ CD11b+) with further expression of MPO, arginase 1, PD-L1, CD10, or CD16 and **B)** MDSC subpopulations (M-MDSC: CD14+ CD15- CD11b+ HLA-DR-, PMN-MDSC: CD14-CD15+ CD11b+ HLA-DR-, e-MDSC: CD14- CD15- HLA-DR- CD33+) expressing PD-L1 are exemplarily shown on a blood sample (B [top, e-MDSC, M-MDSC]) or a tumor tissue sample (A, B [HLA-DR, CD15, PMN-MDSC, PD-L1]).


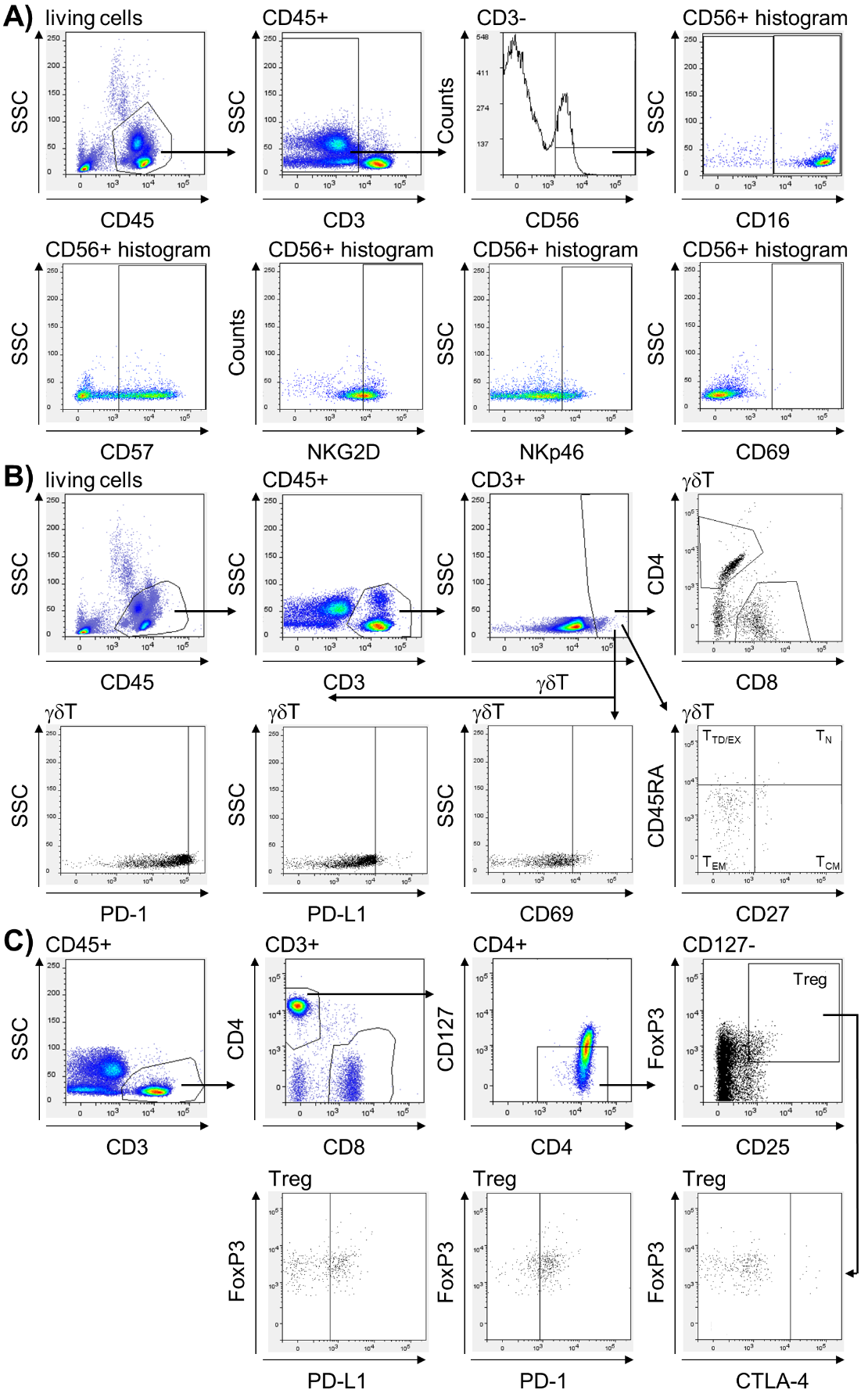


**Supplementary Figure 3. Exemplary gating strategies of lymphoid immune cell populations.** Gating strategies of **A)** CD56+ NK cells as well as further surface receptors and maturation/activation markers (CD16, CD57, NKG2D, NKp46, CD69), **B)** γδT cells with respective expression of CD4, CD8, PD-1, PD-L1, CD69 or differentiation stages and **C)** T_reg_ (CD4+ FoxP3+ CD25+ CD127-) expressing PD-L1, PD-1 or CTLA-4 are exemplarily shown on a blood sample (A, C [top]) or an ascites sample (B, C [bottom]).


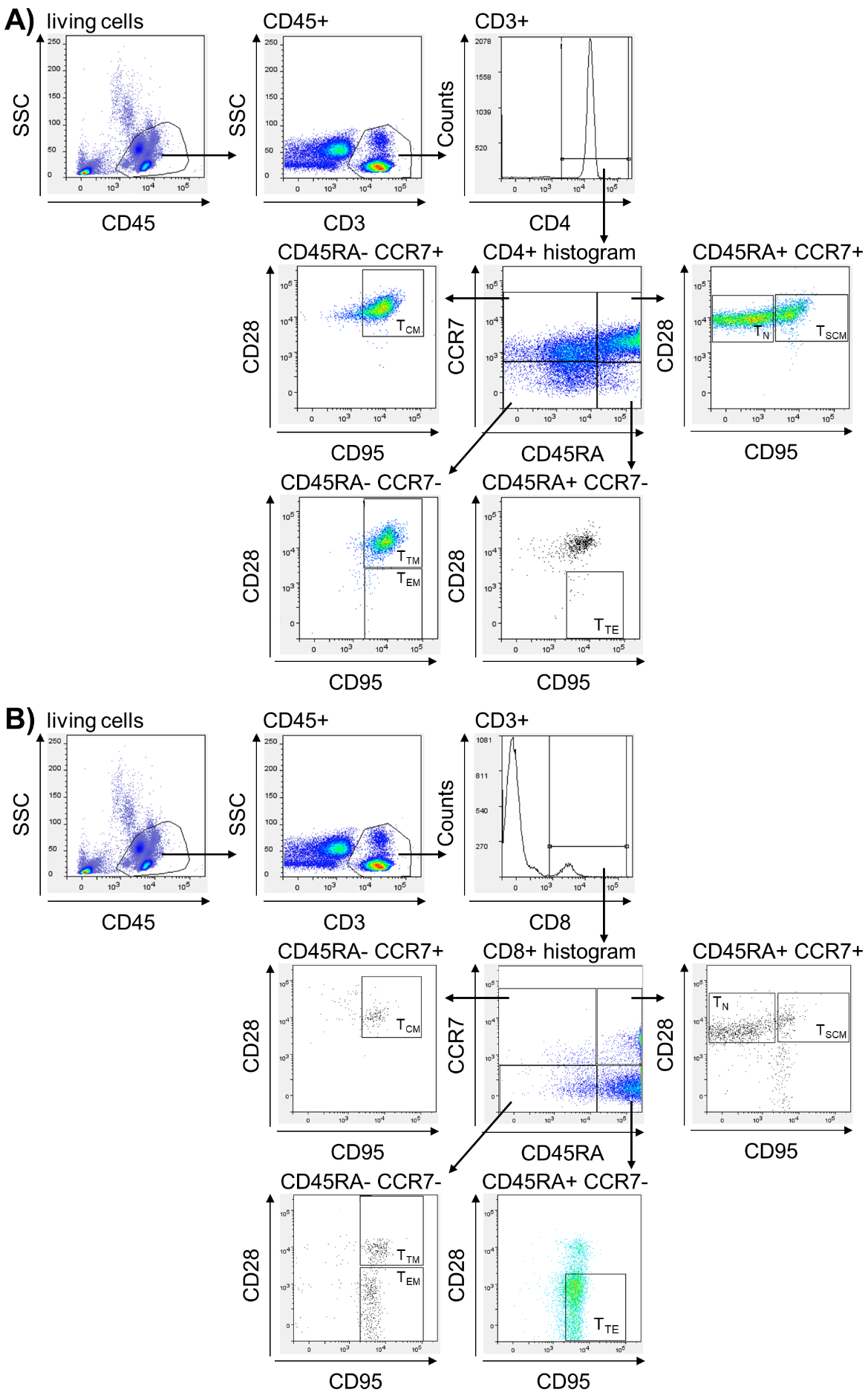


**Supplementary Figure 4. Exemplary gating strategies of CD4+ and CD8+ T cell subpopulations. Gating strategies of A)** CD4+ T cells and **B)** CD8+ T cells with respect to their differentiation stages are exemplarily shown on blood samples.


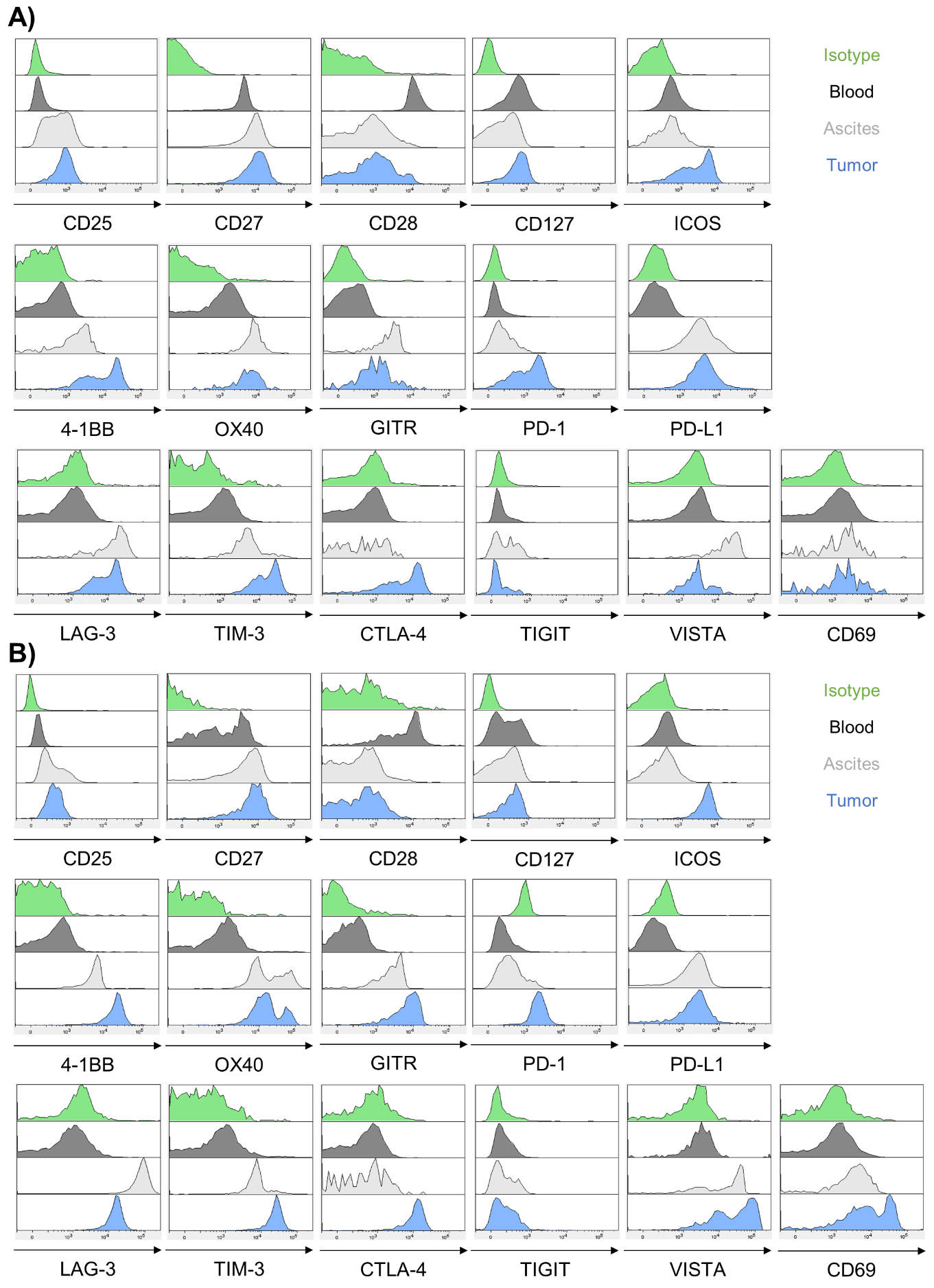


**Supplementary Figure 5. Exemplary histograms of T cell populations expressing marker molecules.** Histograms of expressed surface markers exemplarily shown for **A)** CD4+ T cells and **B)** CD8+ T cells in all investigated compartments compared to corresponding isotype controls.


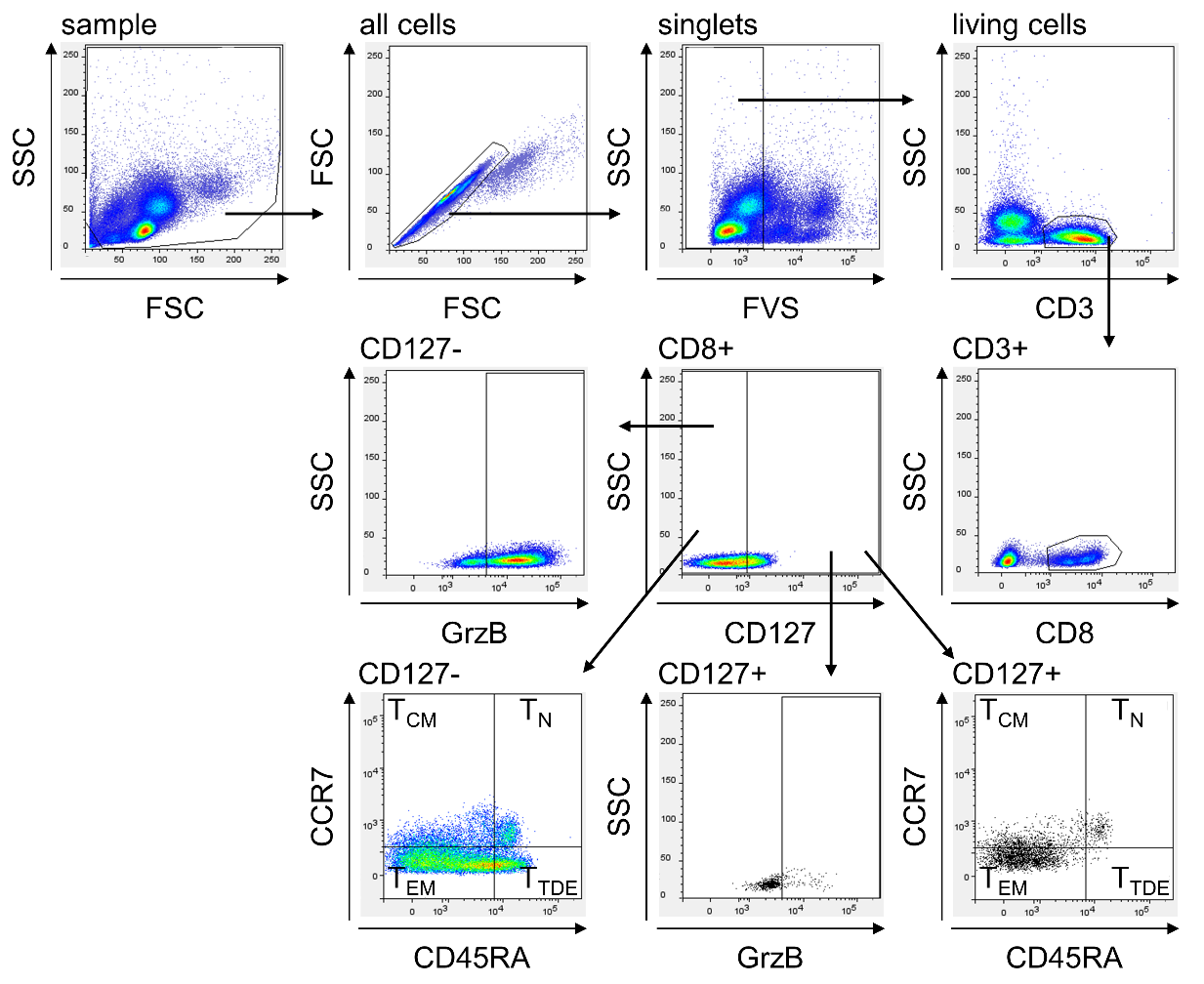


**Supplementary Figure 6. Exemplary gating strategies of CD127+ CD8+ and CD127- CD8+ T cell subpopulations.** Gating strategies of CD127**+** CD8+ T cells and corresponding CD127- CD8+ T cells with respect to granzyme B expression (GrzB) and their differentiation stages (T_N_, T_CM_, T_EM_, T_TDE_) are exemplarily shown on a blood sample.


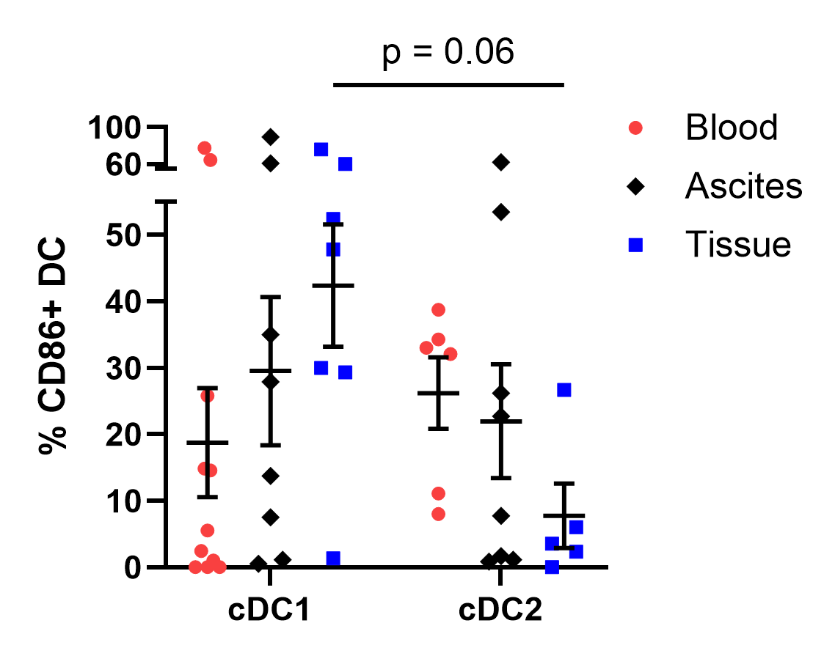


**Supplementary Figure 7. Comparison of CD141+ cDC1 and CD1c+ cDC2 in matched peripheral blood, ascites, and tumor samples regarding their CD86+ expression.** Mean ± SEM, two-way ANOVA, Bonferroni post-hoc test.


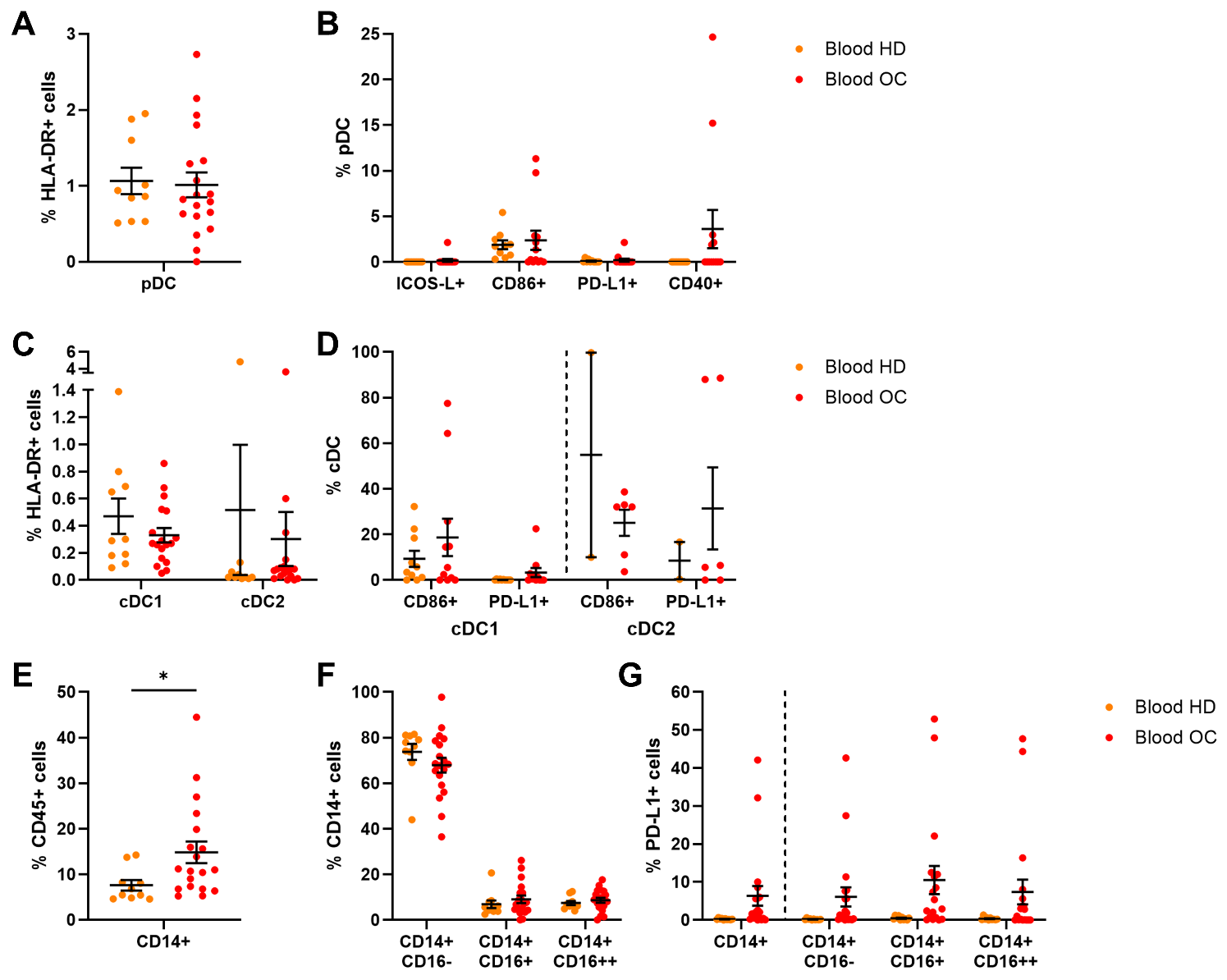


**Supplementary Figure 8. Characterization of DC and monocyte subpopulations in peripheral blood of ovarian cancer (OC) patients and healthy donors (HD, gender- and age-matched).** Dot plots show the proportions of A) pDC and B) their expressed markers as well as C) cDC1, cDC2, and D) the analyzed marker molecules. The proportion of E) monocytes, F) monocyte subsets according to CD16 expression, and G) their PD-L1 expression is presented. Mean ± SEM, unpaired t-test, * p ≤ 0.05.


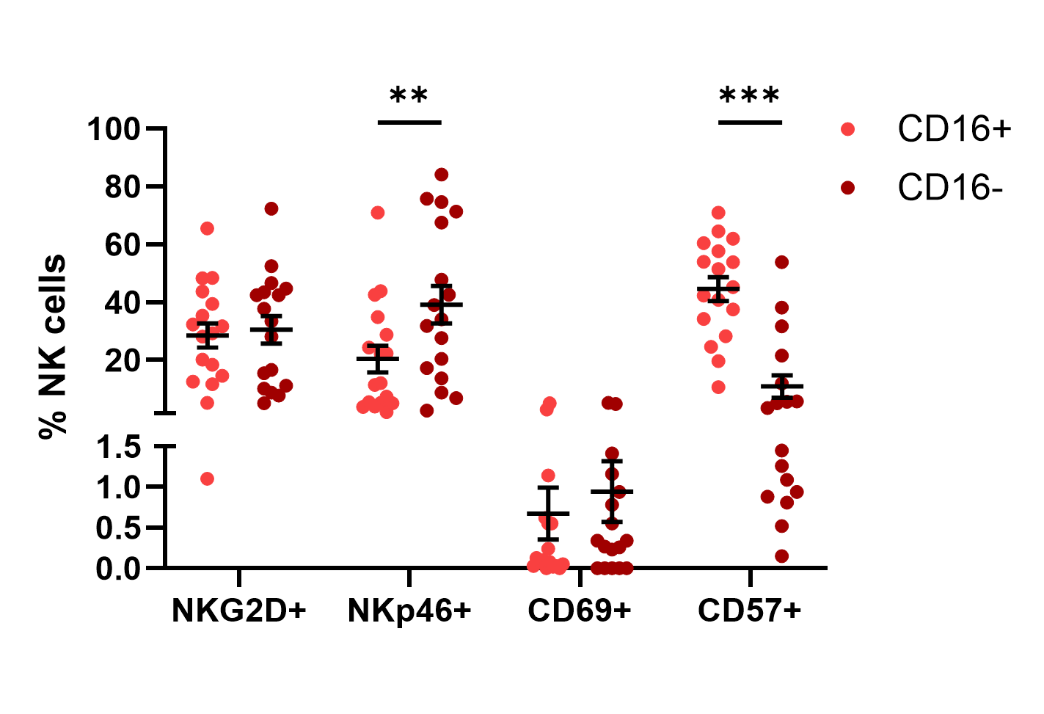


**Supplementary Figure 9. Comparison of CD16+ and CD16- NK cells in matched peripheral blood samples regarding their expressed markers (NKG2D, NKp46, CD69, CD57).** Mean ± SEM, two-way ANOVA, Bonferroni post-hoc test, ** p ≤ 0.01, *** p < 0.001.


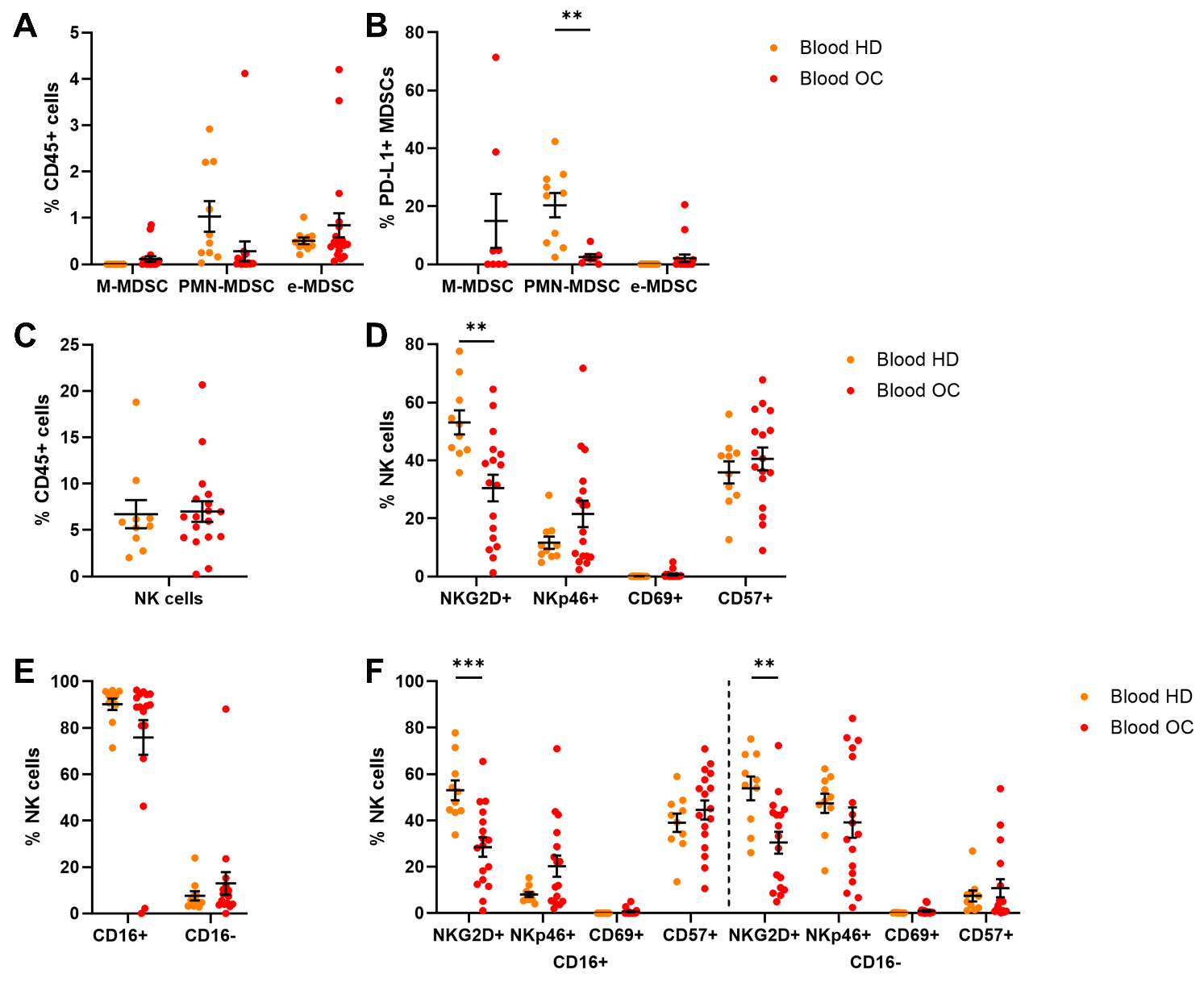


**Supplementary Figure 10.** **Characterization of MDSC and NK cells in peripheral blood of ovarian cancer (OC) patients and healthy donors (HD, gender- and age-matched).** Dot plots show the proportion of **A)** MDSC subsets and **B)** their PD-L1 expression. Similarly, the frequencies of **E)** NK cells and **F)** their marker molecules expressed as well as **G)** the proportion of CD16+ and CD16- NK cell subpopulations and **H)** their expressed markers are presented. Mean ± SEM, unpaired t-test, ** p ≤ 0.01, *** p < 0.001.


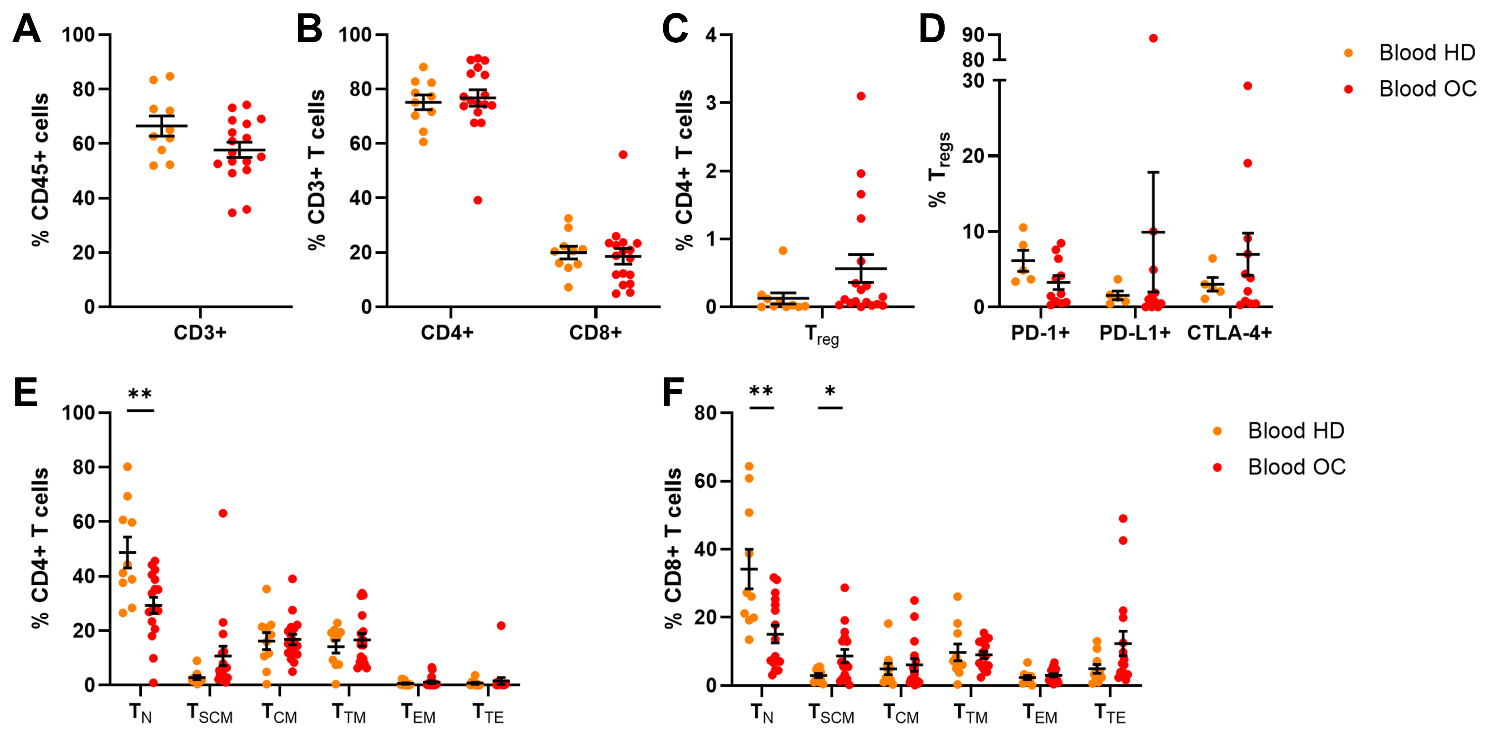


**Supplementary Figure 11. Distribution and maturation of αβT cell subpopulations in** **peripheral blood of ovarian cancer (OC) patients and healthy donors (HD, gender- and age-matched).** Dot plots show the proportions of **A)** CD3+ T cells, **B)** CD4+ and CD8+ αβT cells, **C)** T_reg_ and **D)** T_reg_ inhibitory marker molecules, as well as **E)** CD4+ T cell and **F)** CD8+ T cell differentiation stages. Mean ± SEM, unpaired t-test, * p ≤ 0.05, ** p ≤ 0.01.


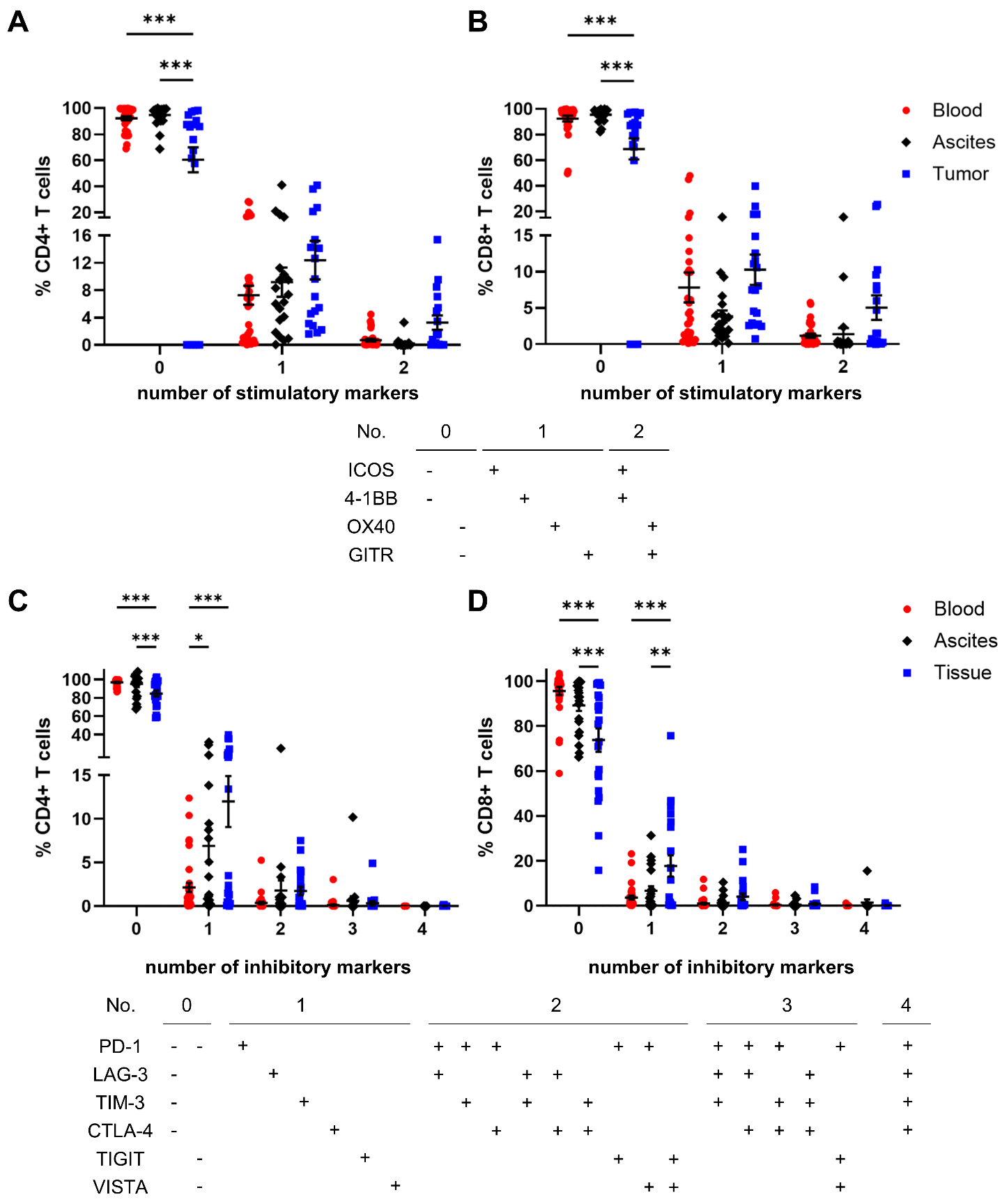


**Supplementary Figure 12. Receptor expression profiling of T cells in matched peripheral blood, ascites, and tumor tissue samples.** Proportion of CD4+ (left) and CD8+ (right) T cells expressing 0, 1, or 2 co-activation marker combinations **(A, B)** and 0, 1, 2, 3, or 4 co-inhibitory **(C, D)** consolidated as indicated for the individual marker proteins below the graphs. Marker combinations were calculated by Boolean gating in FlowLogic^TM^ using AND function for column-wise and XOR function for row-wise linkage in case of positive co-expression as well as AND NOT function for absent receptor expression as shown in tables. Mean ± SEM, two-way ANOVA, Bonferroni post-hoc test, * p ≤ 0.05, ** p ≤ 0.01, *** p < 0.001.


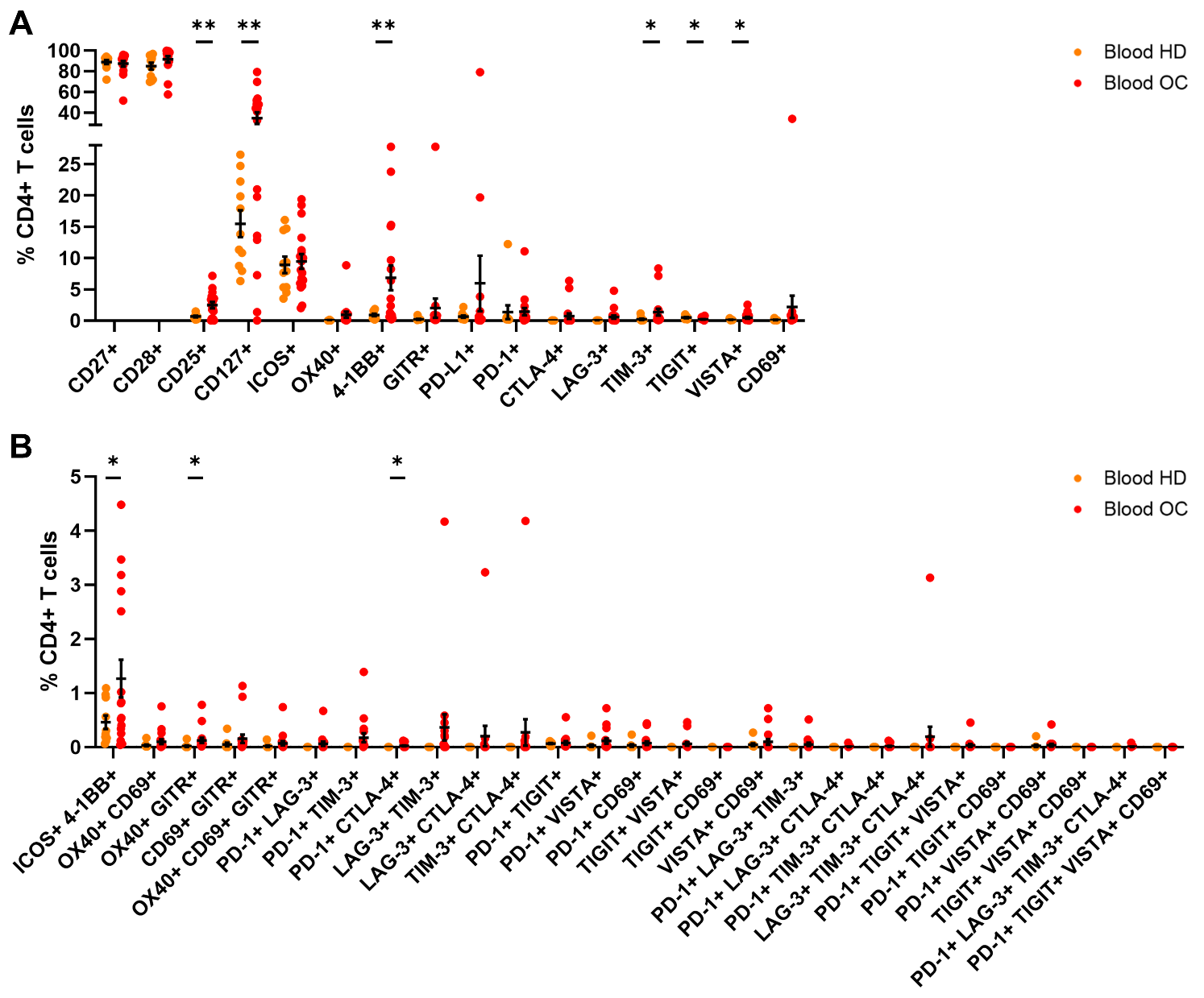


**Supplementary Figure 13. Receptor expression profiling of CD4+ T cells in peripheral blood of ovarian cancer (OC) patients and healthy donors (HD, gender- and age-matched).** Proportions of CD4+ T cells expressing **A)** single markers are shown in dot plots as well as **B)** proportions of CD4+ T cells expressing two to four receptor combinations calculated by Boolean gating in FlowLogic^TM^ using AND function. Mean ± SEM, unpaired t-test, * p ≤ 0.05, ** p ≤ 0.01.


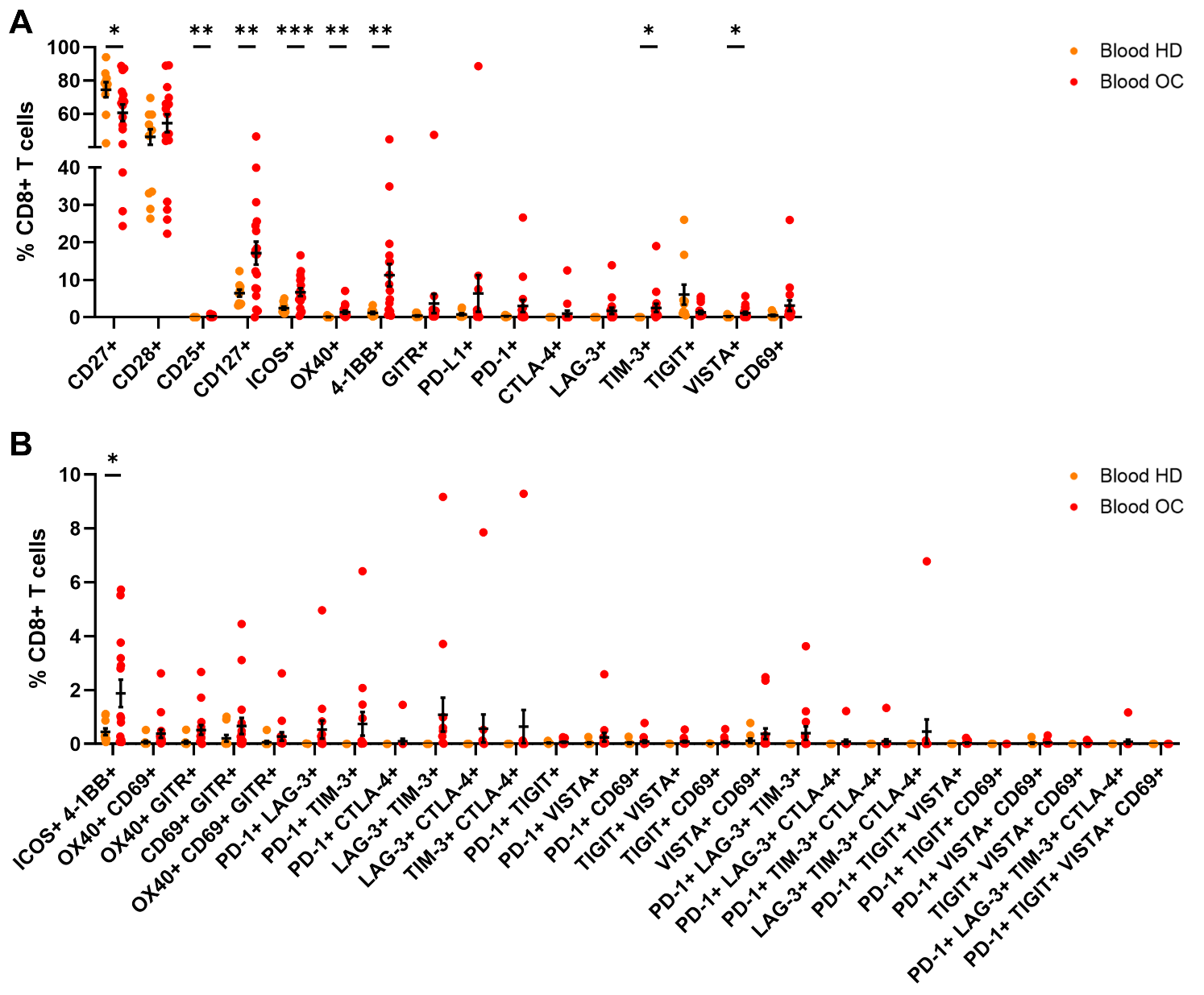


**Supplementary Figure 14. Receptor expression profiling of CD8+ T cells** **in peripheral blood of ovarian cancer (OC) patients and healthy donors (HD, gender- and age-matched).** **A)** Dot plots present proportions of CD8+ T cells expressing single markers. **B)** Proportions of CD8+ T cells expressing two to four receptor combinations calculated by Boolean gating in FlowLogic^TM^ using AND function are shown. Mean ± SEM, unpaired t-test, * p ≤ 0.05, ** p ≤ 0.01, *** p ≤ 0.001.


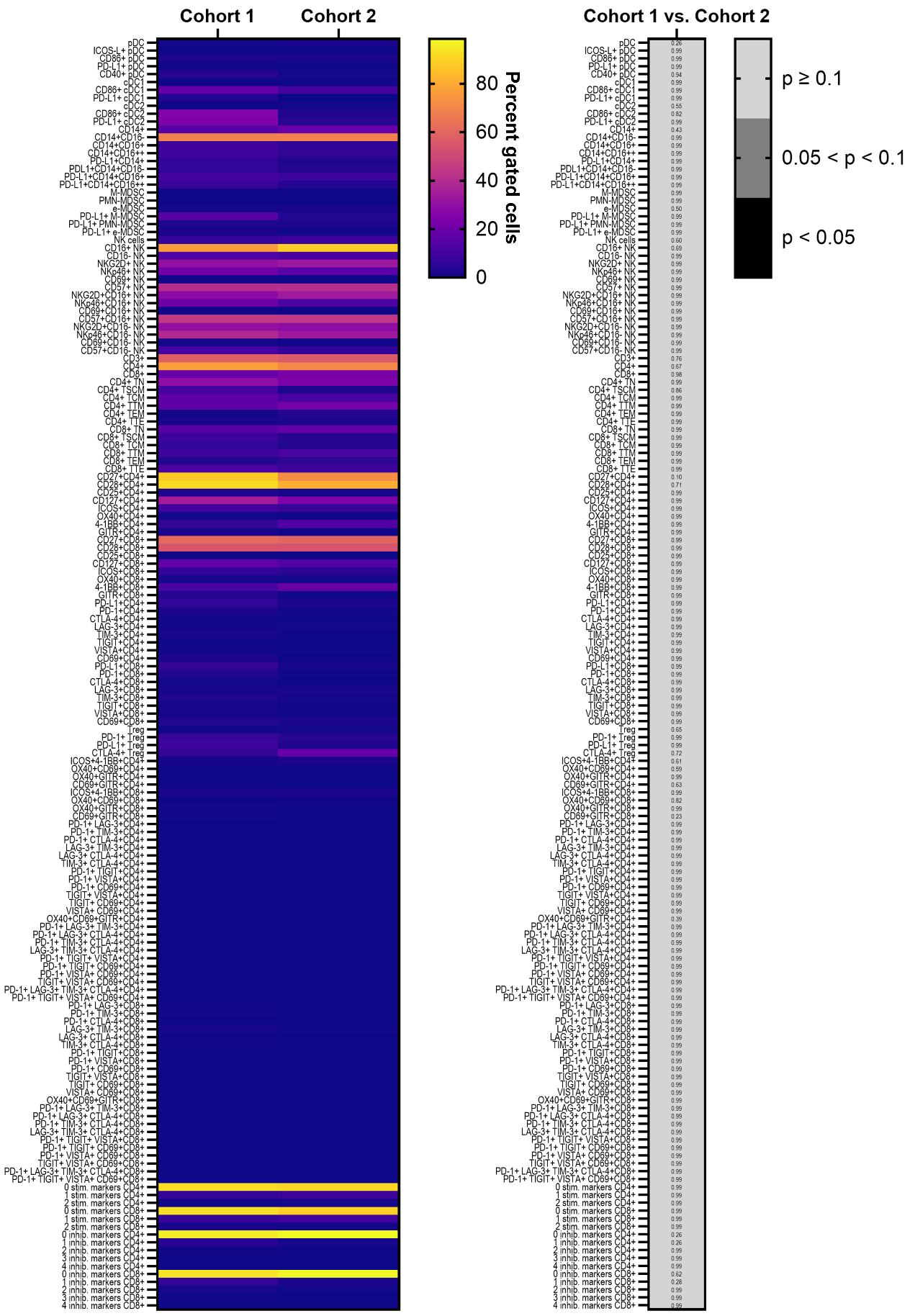


**Supplementary Figure 15. Comparison of investigated immune cell phenotypes in peripheral blood samples of matched patients (main cohort, cohort 1) with additional patients (extended cohort, cohort 2).** Left: Mean immune cell frequencies in % positive gated cells; Right: p-values calculated by unpaired t-test or two-way ANOVA with Bonferroni post-hoc test.


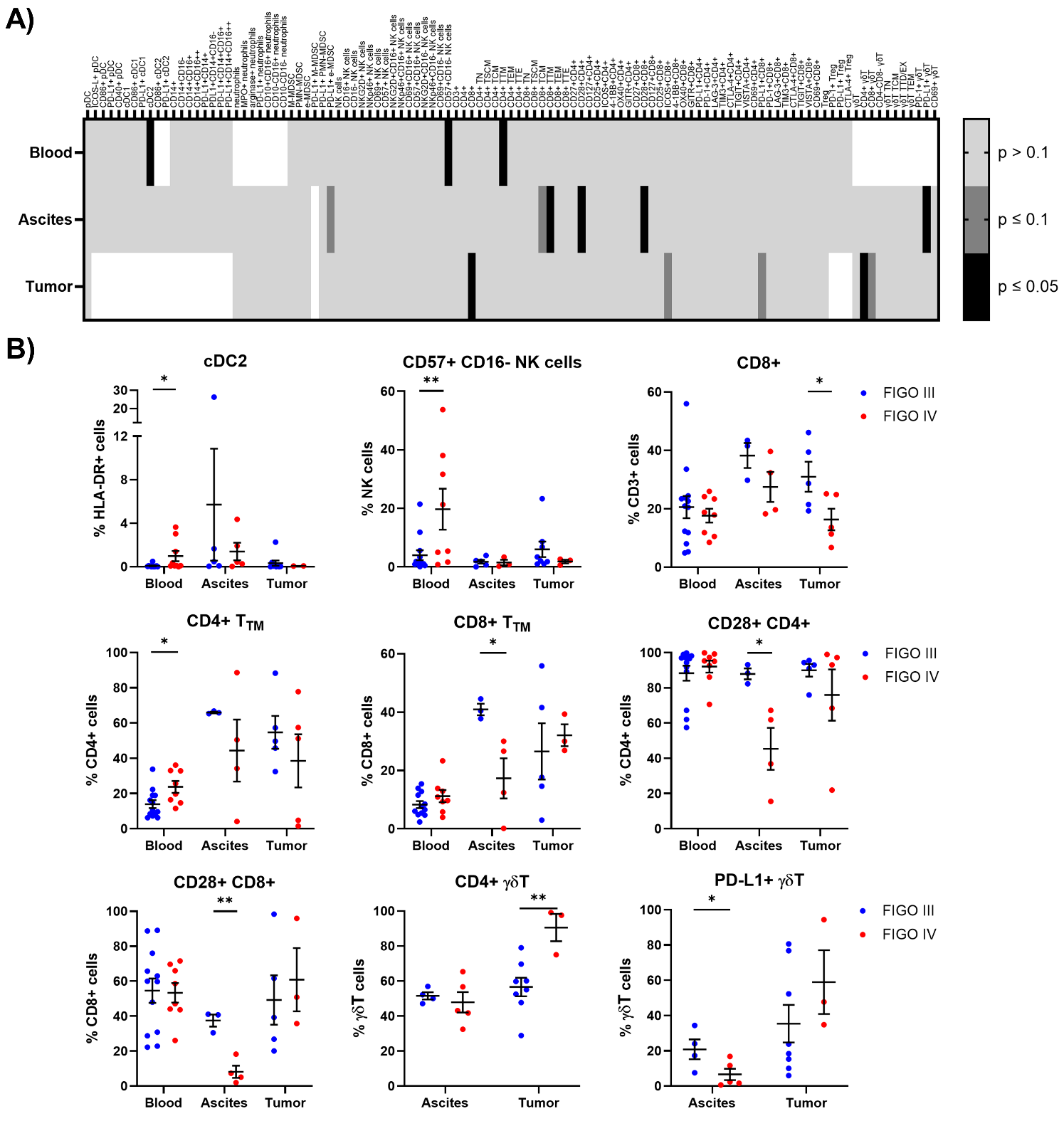


**Supplementary Figure 16. Association of immune cells in peripheral blood, ascites, and tumor tissue samples with FIGO stage of high-grade serous OC patients. A)** p-values are presented in a heatmap-like manner for individual immune cell phenotypes in the investigated compartments between FIGO III and IV. Comparisons with less than three datapoints in each group were excluded. **B)** Significant differences (p ≤ 0.05) of immune cell phenotypes in at least one compartment are depicted separately as dot plots for FIGO stage III vs. FIGO stage IV. Mean ± SEM, unpaired t-test, * p ≤ 0.05, ** p ≤ 0.01.


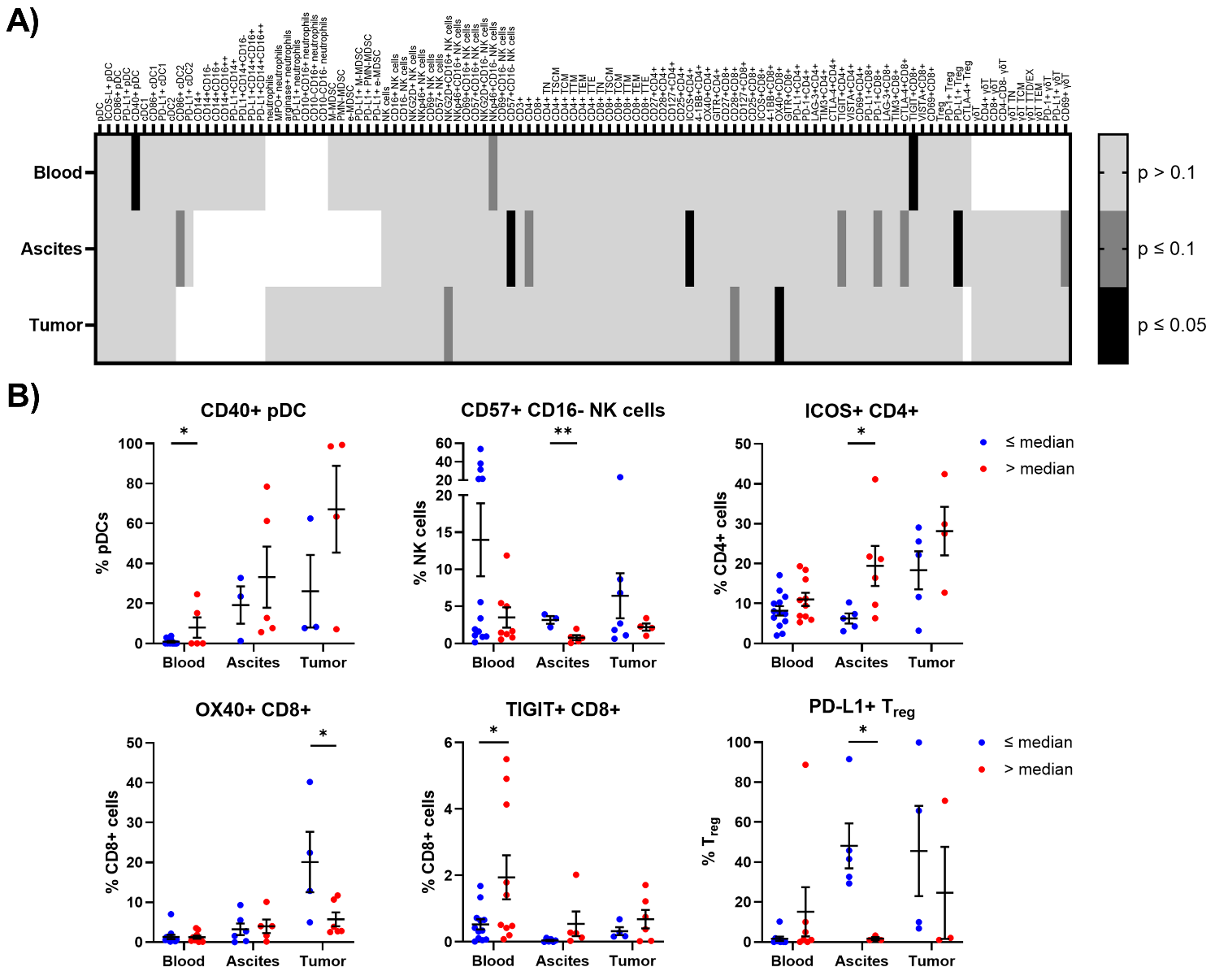


**Supplementary Figure 17. Association of immune cells in peripheral blood, ascites, and tumor tissue samples with serum CA125 value of high-grade serous OC patients. A)** Heatmap-like graph shows p-values for individual immune cell phenotypes in the investigated compartments between CA125 groups classified according to median serum CA125 value of the whole cohort (CA125 median = 487.5 IU/ml). Comparisons with less than three datapoints in each group were excluded. **B)** Significant differences (p ≤ 0.05) of immune cell phenotypes in at least one compartment are depicted separately as dot plots for CA125 groups. Mean ± SEM, unpaired t-test, * p ≤ 0.05, ** p ≤ 0.01.


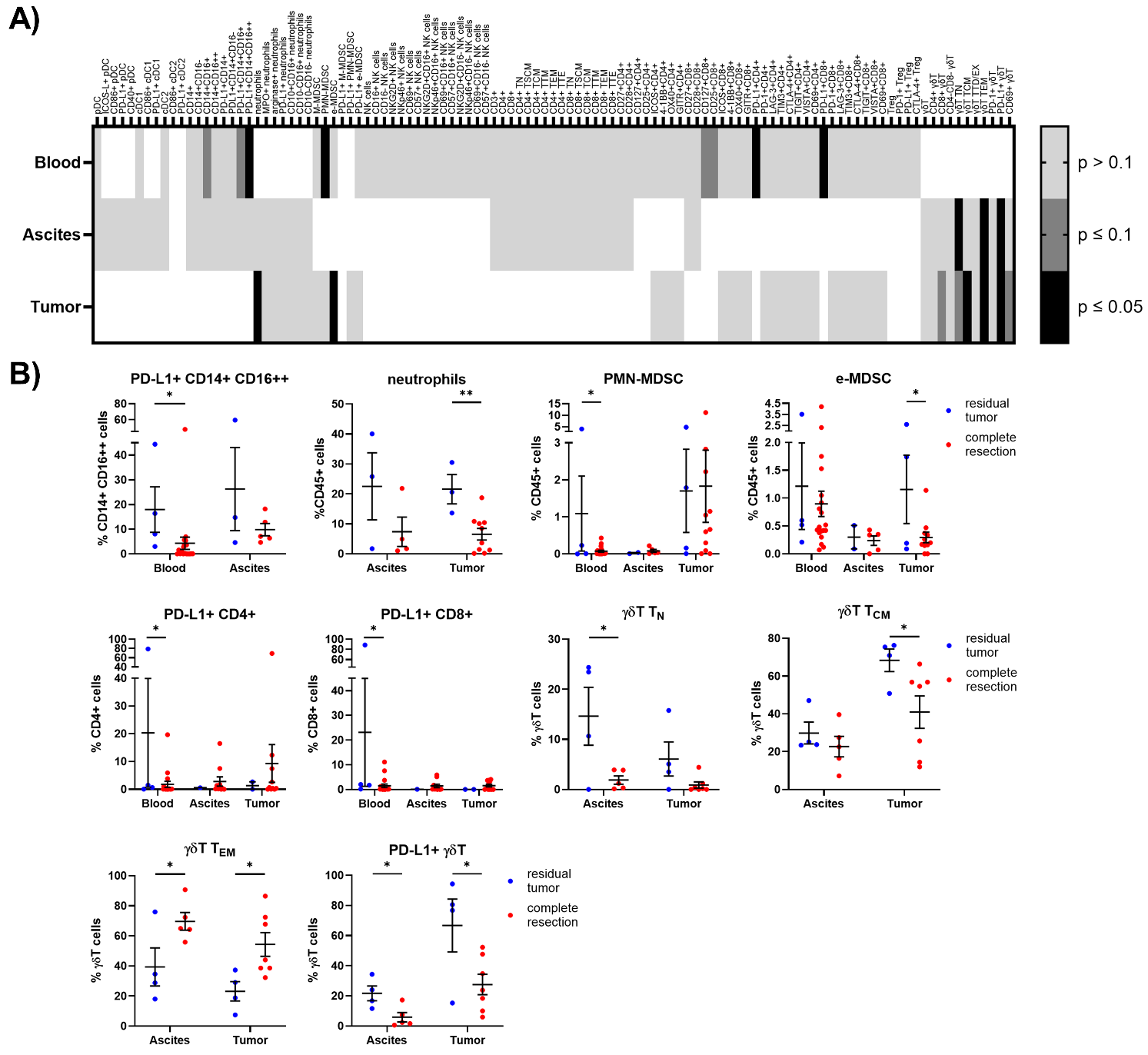


**Supplementary Figure 18. Association of immune cells in peripheral blood, ascites, and tumor tissue samples with surgical outcome of high-grade serous OC patients. A)** Heatmap-like graph shows p-values for individual immune cell phenotypes in the investigated compartments between OC patients with macroscopic complete resection and residual tumor after surgery. Comparisons with less than three datapoints in each group were excluded. **B)** Significant differences (p ≤ 0.05) of immune cell phenotypes in at least one compartment are depicted separately as dot plots for OC patients stratified according to their surgical outcome. Mean ± SEM, unpaired t-test, * p ≤ 0.05, ** p ≤ 0.01.


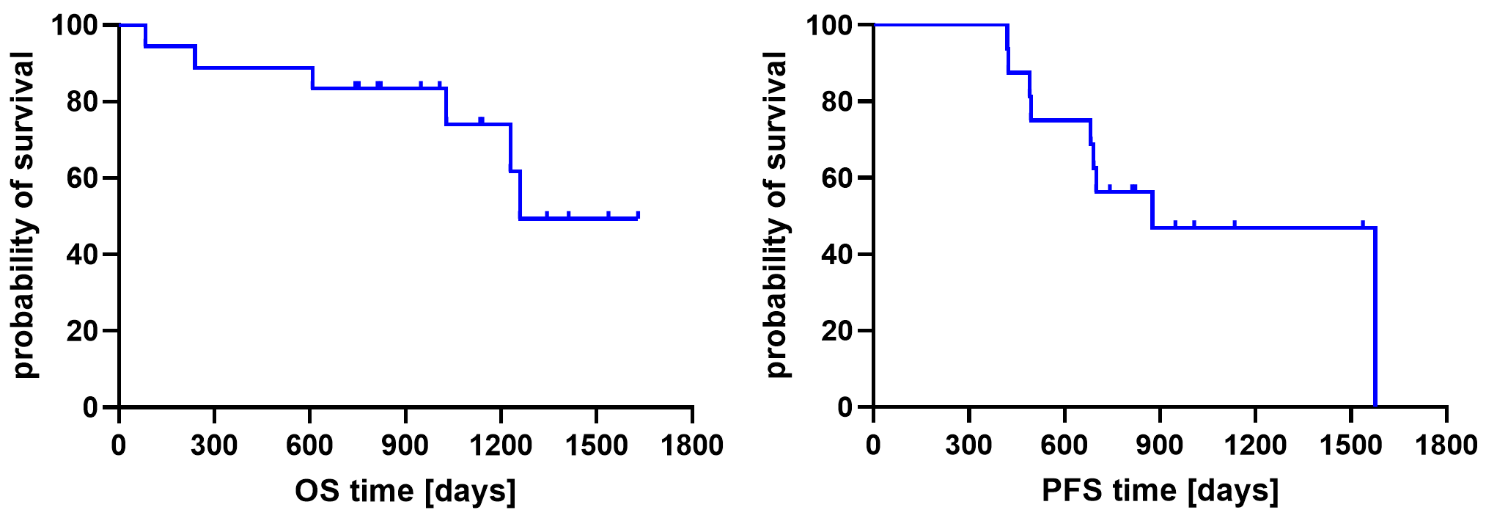


**Supplementary Figure 19. Overall survival (OS, left) and progression-free survival (PFS, right) of high-grade serous OC patients with a follow-up period of at least two years.**


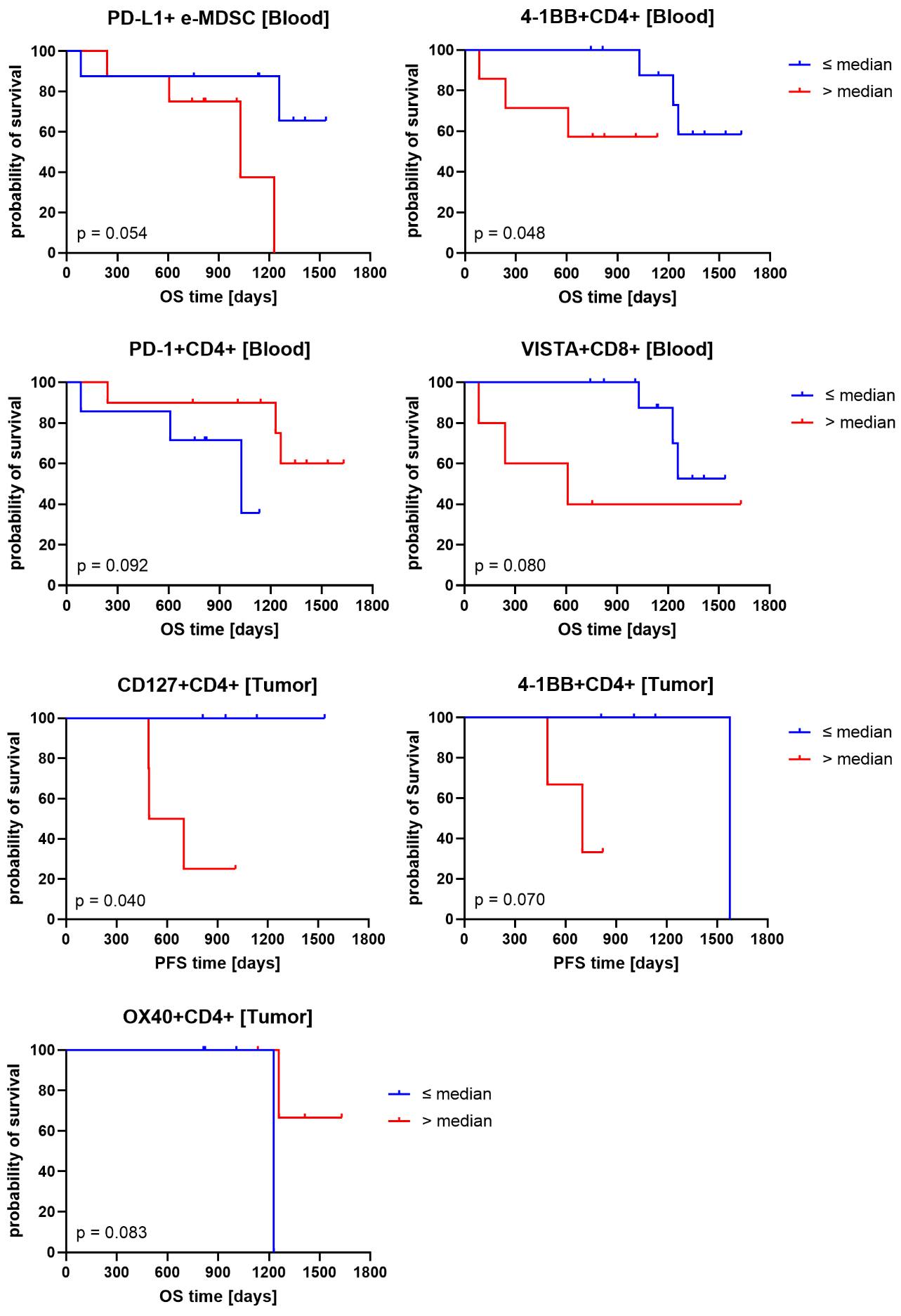


**Supplementary Figure 20. Overall survival (OS) and progression-free survival (PFS) of high-grade serous OC patients separated according to median frequencies of the respective immune cell markers in peripheral blood or matched tumor samples.** p-values calculated by Log-rank test.


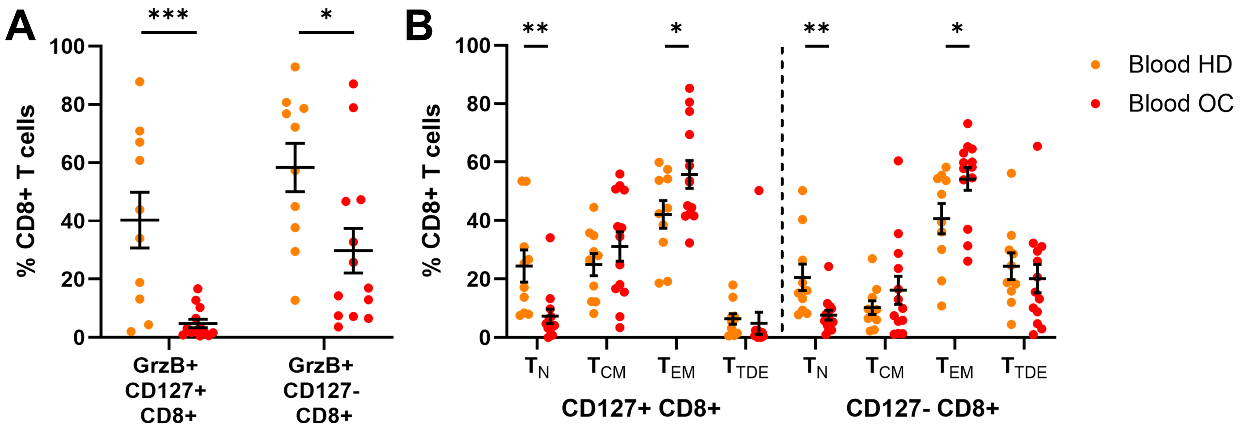


**Supplementary Figure 21. Granzyme B (GrzB) expression of CD127+ CD8+ T cells in peripheral blood samples of ovarian cancer (OC) patients and healthy donors (HD, gender- and age-matched).** Flow cytometric analysis of 13 frozen (OC) and 10 fresh PBMC samples (HD) isolated from PB with respect to **A)** GrzB expression of CD127+ CD8+ T cells and CD127- CD8+ T cells as well as **B)** maturation stages of CD127**+** CD8+ T cells and their CD127- counterparts after 4h incubation with PMA, ionomycin, and brefeldin A. Mean ± SEM, unpaired t-test, * p ≤ 0.05, ** p ≤ 0.01, *** p < 0.001.

# Supplementary Tables

Supplementary Table 1: Antibodies for extracellular and intracellular (*) flow cytometric staining of analyzed immune cell marker panels.

| **Antigen (clone)** | **Company** | **RRID** | **Conjugate** | **Isotype control (clone)** | **Company** |
| --- | --- | --- | --- | --- | --- |
| 4-1BB (4B4-1) | BD Biosciences | AB_2742840 | BV605 | Mouse IgG1 (MOPC-21) | Biolegend |
| Arginase 1* (658922) | R&D Systems | AB_3656659 | PE | Mouse IgG2b (MG2b-57) | Biolegend |
| CCR7 (150503) | BD Biosciences | AB_2259847 | FITC | Mouse IgG2a (MOPC-173) | Biolegend |
| CCR7 (3D12) | BD Biosciences | AB_396765 | PE-Cy7 | Rat IgG2a (RTK2758) | Biolegend |
| CD1c (L161) | Biolegend | AB_2563657 | AF700 | Mouse IgG1 (MOPC-21) | Biolegend |
| CD3 (UCHT1) | BD Biosciences | AB_11152082 | BV421 | Mouse IgG1 (MOPC-21) | Biolegend |
| CD3 (SK7) | BD Biosciences | AB_1645475 | APC-H7 | Mouse IgG1 (X40) | BD Biosciences |
| CD3 (HIT3a) | BD Biosciences | AB_395745 | FITC | Mouse IgG2a (MOPC-173) | Biolegend |
| CD4 (RPA-T4) | BD Biosciences | AB_396943 | AF700 | Mouse IgG1 (MOPC-21) | Biolegend |
| CD4 (RPA-T4) | BD Biosciences | AB_397037 | PB | Mouse IgG1 (MOPC-21) | Biolegend |
| CD8 (RPA-T8) | BD Biosciences | AB_397058 | PB | Mouse IgG1 (MOPC-21) | Biolegend |
| CD8 (SK1) | BD Biosciences | AB_10896281 | V500 | Mouse IgG1 (X40) | BD Biosciences |
| CD10 (HI10A) | BD Biosciences | AB_11154416 | PE-CF594 | Mouse IgG1 (MOPC-21) | Biolegend |
| CD11b (ICFR44) | BD Biosciences | AB_396860 | APC-Cy7 | Mouse IgG1 (X40) | BD Biosciences |
| CD11c (B-ly6) | BD Biosciences | AB_2744276 | BV605 | Mouse IgG1 (MOPC-21) | Biolegend |
| CD11c (B-ly6) | BD Biosciences | AB_398680 | APC | Mouse IgG1 (MOPC-21) | Biolegend |
| CD14 (M5E2) | Biolegend | AB_2563798 | BV605 | Mouse IgG2a (MOPC-173) | Biolegend |
| CD14 (MφP9) | BD Biosciences | AB_2737729 | PE-Cy7 | Mouse IgG2b (27-35) | BD Biosciences |
| CD15 (HI98) | BD Biosciences | AB_398501 | APC | Mouse IgM (MOPC-21) | Biolegend |
| CD16 (3G8) | BD Biosciences | AB_396941 | AF700 | Mouse IgG1 (MOPC-21) | Biolegend |
| CD19 (HIB19) | BD Biosciences | AB_1727437 | APC-H7 | Mouse IgG1 (X40) | BD Biosciences |
| CD19 (HIB19) | BD Biosciences | AB_11153299 | BV421 | Mouse IgG1 (MOPC-21) | Biolegend |
| CD19 (HIB19) | BD Biosciences | AB_395812 | FITC | Mouse IgG1 (MOPC-21) | Biolegend |
| CD25 (M-A251) | BD Biosciences | AB_395825 | FITC | Mouse IgG1 (MOPC-21) | Biolegend |
| CD25 (M-A251) | BD Biosciences | AB_396847 | PE-Cy7 | Mouse IgG1 (MOPC-21) | Biolegend |
| CD27 (M-T271) | BD Biosciences | AB_395833 | FITC | Mouse IgG1 (MOPC-21) | Biolegend |
| CD27 (O323) | Biolegend | AB_2561450 | BV605 | Mouse IgG1 (MOPC-21) | Biolegend |
| CD28 (CD28.2) | BD Biosciences | AB_1727459 | PE-Cy7 | Mouse IgG1 (MOPC-21) | Biolegend |
| CD28 (CD28.2) | BD Biosciences | AB_398666 | APC | Mouse IgG1 (MOPC-21) | Biolegend |
| CD33 (P67.6) | Biolegend | AB_2566107 | PE | Mouse IgG1 (MOPC-21) | Biolegend |
| CD40 (5C3) | Biolegend | AB_2564244 | BV605 | Mouse IgG1 (MOPC-21) | Biolegend |
| CD40 (5C3) | BD Biosciences | AB_395964 | PE | Mouse IgG1 (MOPC-21) | Biolegend |
| CD45 (HI30) | BD Biosciences | AB_1645573 | V450 | Mouse IgG1 (MOPC-21) | Biolegend |
| CD45 (HI30) | BD Biosciences | AB_395875 | PE | Mouse IgG1 (MOPC-21) | Biolegend |
| CD45 (HI30) | BD Biosciences | AB_1937324 | V500 | Mouse IgG1 (X40) | BD Biosciences |
| CD45 (HI30) | BD Biosciences | AB_1645452 | AF700 | Mouse IgG1 (MOPC-21) | Biolegend |
| CD45 (DX2) | BD Biosciences | AB_11153666 | PE-CF594 | Mouse IgG1 (MOPC-21) | Biolegend |
| CD45RA (H100) | BD Biosciences | AB_395880 | PE | Mouse IgG2b (MPC-11) | Biolegend |
| CD45RA (HI100) | BD Biosciences | AB_1727496 | AF700 | Mouse IgG2b (MPC-11) | Biolegend |
| CD56 (B159) | BD Biosciences | AB_398601 | APC | Mouse IgG1 (MOPC-21) | Biolegend |
| CD56 (B159) | BD Biosciences | AB_2737799 | FITC | Mouse IgG1 (MOPC-21) | Biolegend |
| CD57 (NK-1) | BD Biosciences | AB_395986 | FITC | Mouse IgM (MM-30) | Biolegend |
| CD66b (G10F5) | Biolegend | AB_2566605 | PE-Cy7 | Mouse IgM (MM-30) | Biolegend |
| CD69 (L78) | BD Biosciences | AB_400523 | APC | Mouse IgG1 (MOPC-21) | Biolegend |
| CD69 (FN50) | BD Biosciences | AB_2687422 | BV421 | Mouse IgG1 (X40) | BD Biosciences |
| CD86 (2331 FUN-1) | BD Biosciences | AB_396012 | FITC | Mouse IgG1 (MOPC-21) | Biolegend |
| CD86 (2331 FUN-1) | BD Biosciences | AB_10563077 | PE-Cy7 | Mouse IgG1 (MOPC-21) | Biolegend |
| CD95 (DX2) | BD Biosciences | AB_398659 | APC | Mouse IgG1 (MOPC-21) | Biolegend |
| CD95 (DX2) | BD Biosciences | AB_11153666 | Pe-CF594 | Mouse IgG1 (MOPC-21) | Biolegend |
| CD123 (6H6) | Biolegend | AB_493576 | PE-Cy7 | Mouse IgG1 (MOPC-21) | Biolegend |
| CD127 (HIL-7R-M21) | BD Biosciences | AB_1645486 | FITC | Mouse IgG1 (MOPC-21) | Biolegend |
| CD127 (HIL-7R-M21) | BD Biosciences | AB_2033938 | PE-Cy7 | Mouse IgG1 (MOPC-21) | Biolegend |
| CD127 (HIL-7R-M21) | BD Biosciences | AB_1645548 | PerCP-Cy5.5 | Mouse IgG1 (MOPC-21) | Biolegend |
| CD141 (AD5-14H12) | Miltenyi | AB_2726095 | PE | Mouse IgG1 (MOPC-21) | Biolegend |
| CD303 (AC144) | Miltenyi | AB_2726015 | APC | Mouse IgG1 (MOPC-21) | Biolegend |
| CTLA-4 (L3D10) | Biolegend | AB_10679122 | APC | Mouse IgG1 (MOPC-21) | Biolegend |
| CTLA-4 (L3D10) | Biolegend | AB_2566198 | PE-CF594 | Mouse IgG1 (MOPC-21) | Biolegend |
| FoxP3 * (259D/C7) | BD Biosciences | AB_1645508 | PE | Mouse IgG1 (MOPC-21) | Biolegend |
| FoxP3 * (PCH101) | Invitrogen | AB_10804638 | PE-Cy7 | Rat IgG2a (RTK2758) | Biolegend |
| γδTCR (B1) | Biolegend | AB_2562891 | PE-Cy7 | Mouse IgG1 (MOPC-21) | Biolegend |
| GITR (108-17) | Biolegend | AB_2687163 | BV605 | Mouse IgG2a (MOPC-173) | Biolegend |
| GrzB * (QA16A02) | Biolegend | AB_2687028 | APC | Mouse IgG1 (MOPC-21) | Biolegend |
| HLA-DR (L243) | BD Biosciences | AB_2870307 | APC-H7 | Mouse IgG2a (G155-178) | BD Biosciences |
| HLA-DR (G46-6) | BD Biosciences | AB_10563765 | V500 | Mouse IgG2a (G155-178) | BD Biosciences |
| ICOS (C398.4A) | Biolegend | AB_416330 | FITC | Armenian Hamster IgG (HTK888) | Biolegend |
| ICOS-L (2D3/B7-H2) | BD Biosciences | AB_394404 | PE | Mouse IgG2b (MPC-11) | Biolegend |
| LAG-3 (polyclonal) | R&D Systems | AB_2133351 | PE | Goat IgG (POLY24030) | Biolegend |
| MPO * (MPO-7) | Dako | AB_2917957 | FITC | Mouse IgG1 (MOPC-21) | Biolegend |
| NKG2D (ON72) | Beckman Coulter | AB_2801262 | PE | Mouse IgG1 (MOPC-21) | Biolegend |
| NKp46 (9E2/NKp46) | BD Biosciences | AB_10894195 | PE-Cy7 | Mouse IgG1 (MOPC-21) | Biolegend |
| OX40 (Ber-ACT35) | Biolegend | AB_10901161 | PE-Cy7 | Mouse IgG1 (MOPC-21) | Biolegend |
| PD-1 (EH12.2H7) | Biolegend | AB_940475 | APC | Mouse IgG1 (MOPC-21) | Biolegend |
| PD-1 (EH12.1) | BD Biosciences | AB_2738091 | BV605 | Mouse IgG1 (MOPC-21) | Biolegend |
| PD-1 (EH12.1) | BD Biosciences | AB_2033989 | PE | Mouse IgG1 (MOPC-21) | Biolegend |
| PD-1 (EH12.2H7) | Biolegend | AB_2562256 | BV510 | Mouse IgG1 (MOPC-21) | Biolegend |
| PD-L1 (MIH1) | BD Biosciences | AB_647198 | PE | Mouse IgG1 (MOPC-21) | Biolegend |
| PD-L1 (MIH1) | BD Biosciences | AB_2740156 | BV605 | Mouse IgG1 (MOPC-21) | Biolegend |
| PD-L1 (MIH1) | BD Biosciences | AB_2738399 | APC | Mouse IgG1 (MOPC-21) | Biolegend |
| PD-L1 (MIH1) | BD Biosciences | AB_2739101 | APC-R700 | Mouse IgG1 (X40) | BD Biosciences |
| TIGIT (MBSA43) | Thermo Fisher | AB_2572530 | FITC | Mouse IgG1 (MOPC-21) | Biolegend |
| TIM-3 (F38-2E2) | Biolegend | AB_2561720 | PE-Cy7 | Mouse IgG1 (MOPC-21) | Biolegend |
| VISTA (730804) | R&D Systems | AB_3652241 | PE | Mouse IgG2b (MPC-11) | Biolegend |
